# Comparison of oral cavity protein abundance among caries-free and caries-affected individuals—a systematic review and meta-analysis

**DOI:** 10.3389/froh.2023.1265817

**Published:** 2023-09-15

**Authors:** Eliane Garcia da Silveira, Laura Schaurich Prato, Sarah Freygang Mendes Pilati, Rodrigo Alex Arthur

**Affiliations:** ^1^ Department of Preventive and Community Dentistry, Federal University of Rio Grande do Sul, Porto Alegre, Brazil; ^2^ Faculty of Dentistry, University of Vale do Itajaí, Itajaí, Brazil

**Keywords:** oral cavity, proteome, dental caries, enzymes, markers

## Abstract

**Objective:**

Some salivary proteins seem to be differently abundant among caries-free (CF) and caries-affected (CA) individuals, but previous results are contradictory precluding that definitive conclusion be drawn. A pooled analysis of the available evidence may provide more robust data on identifying oral cavity protein patterns among CF and CA individuals. This systematic review and meta-analysis (PROSPERO CRD42021269079) aimed to compare the oral cavity protein abundance among caries-free and caries-affected individuals.

**Methods:**

This study was conducted following PRISMA guidelines. PubMed, Embase, and Web of Science databases were systematically assessed (up to February 2023) to retrieve clinical studies written in English, German, or in Latin-based languages that compared the oral cavity protein abundance among CF and CA individuals. Data extraction and methodological quality assessment (NIH guidelines) were independently performed by two investigators. Qualitative synthesis was performed from all included studies and meta-analysis was performed using a random-effects model with inverse variance for studies that reported the concentration of proteins or enzymatic activity. Standardized mean difference (SMD) with respective 95% confidence interval (CI) were calculated for each outcome.

**Results:**

A total of 90 studies (two cohort and 88 cross-sectional designs) of more than 6,000 participants were selected for data extraction, being the quality of evidence graded as “fair” for most of them. The oral cavity of CF individuals presented lower total protein concentration [SMD = 0.37 (95% CI: 0.07–0.68; 18 studies)], lower total antioxidant capacity [SMD = 1.29 (95% CI: 0.74–1.85); 17 studies], and lower carbonic anhydrase activity [SMD = 0.83 (95% CI: 0.58–1.09); three studies], whereas CA individuals presented lower carbonic anhydrase concentration [SMD = −0.66 (95% CI: −1.00 to −0.32); three studies], urease [SMD = −0.95 (IC 95%: −1.72 to −0.17); four studies], and arginine deiminase system [SMD = −2.07 (95% CI: −3.53 to −0.62); three studies] activities. Antimicrobial peptides, secretory immunoglobulin-A concentrations and alpha-amylase activity were similar among individuals.

**Conclusion:**

Differences on oral cavity protein abundance were observed among CF and CA individuals. These data indicate some protein patterns for the oral health and dental caries conditions. Even when statistically significant, some of the results were not very consistent. Cohort studies need to be conducted to validate these results.

## 1. Introduction

Dental caries is a plaque-mediated disease that results from a state of dysbiosis induced by frequent exposure to rapidly fermentable sugars (1). In this context, a sugar-rich diet plays a pivotal role on the disease onset (2). This disease inflicts biological, social, and financial negative impacts on the affected individuals as well as it places a high burden on healthcare systems (3). Disease activity should be managed at an individual level at its early stage by means of controlling etiological factors in order to prevent new carious lesions to be developed and to arrest existing ones.

Tooth mineral loss and gain processes are influenced by several factors (4), including the host's saliva (5). Saliva plays an important role on the maintenance of oral health most of its functions, such as the antimicrobial and the pH-buffering functions, the formation of the acquired enamel pellicle, as well as the inhibition of demineralization and the enhancement of remineralization, being played by the salivary proteins/enzymes (6). It is known that saliva comprises of about 2,000 proteins (7, 8). In this context, an important research field, named “Salivaomics,” and the study of the salivary proteome have been increasingly emerged aiming at identifying proteins patterns, which could be considered candidates to estimate the host's risk to the development of oral diseases (9). As the composition of saliva reflects the pathophysiological changes that happen to the human body, it is expected that this biological fluid gives aid to the diagnosis of oral diseases (10). Saliva has numerous advantages as a diagnostic tool: it allows rapid, easy, and non-invasive detection of molecules that could assist on the early diagnosis, on disease progression monitoring, and on its response to therapeutic approaches.

Previous studies have indicated some association between salivary proteins or salivary enzymes and dental caries susceptibility. Caries-affected individuals may present higher total salivary protein concentration (11–13), higher levels of secretory immunoglobulin-A (s-IgA) (14–16), higher concentrations of mucin (17), higher carbonic anhydrase IV (CA-IV) activity (18, 19), higher lysozyme activity (20), higher levels of human beta-defensin (hBD) (21, 22), and lower levels of LL-37 (23), among other differences. However, contradictory results have also been reported (24–37). Moreover, meta-analysis on this subject is still scarce precluding that definitive conclusion be drawn. A pooled analysis of the available evidence may provide more robust data on identifying patterns among caries-free and caries-affected individuals. This way, the aim of this systematic review and meta-analysis was to compare the oral cavity's protein abundance among these individuals in order to identify protein patterns associated with oral health or with dental caries. The tested hypothesis is that the oral cavity protein abundance is different among caries-free and caries-affected individuals.

## 2. Methods

### 2.1. Study protocol

The Preferred Reporting Items for Systematic Review and Meta-Analysis (PRISMA) checklist was used as a guide for conducting and reporting the present study (PRISMA statement) (38). This review was registered in PROSPERO (registry number CRD42021269079). Only data related to protein abundance (expressed as mean and standard deviation or standard error) or electrophoresis-based data were reported in this study.

### 2.2. Review question and PCO strategy

The research question “Does the oral cavity of caries-affected individuals has a different protein abundance compared with the oral cavity of caries-free ones?” was formulated using the Patient, Control, Outcome (PCO) framework (39) as follows: caries-affected individuals (P); caries-free individuals (C); protein abundance (concentration, activity or levels of total proteins, enzymes, immunoglobulins, antioxidants and antimicrobial peptides (AMPs) in dental plaque, saliva or acquired salivary film) (O).

### 2.3. Search strategy and study selection

Electronic search was performed in MEDLINE, Embase, and ISI Web of Science databases without restriction of date of publication. The search strategies were as follows: MEDLINE (biofilm[MeSH Terms]) OR (biofilm) OR (biofilms[MeSH Terms]) OR (biofilms)) OR (dental plaque[MeSH Terms]) OR (dental plaque)) OR (acquired pellicle, salivary[MeSH Terms]) OR (acquired pellicle, salivary) OR (salivary) OR (oral cavity[MeSH Terms]) OR (oral cavity) AND (proteome[MeSH Terms]) OR (proteome) OR (proteomes[MeSH Terms]) OR (proteomes) OR (proteomics[MeSH Terms]) OR (proteomics) AND (dental caries[MeSH Terms]; EMBASE (‘biofilm’/exp OR biofilm OR ‘tooth plaque’/exp OR ‘tooth plaque’ OR ‘dental pellicle’/exp OR ‘dental pellicle’ OR ‘saliva’/exp OR saliva OR ‘mouth cavity’/exp OR 'mouth cavity') AND 'proteome'/exp OR proteome OR 'proteomics’’/exp OR proteomics OR 'protein'/exp) AND 'dental caries’’/exp; ISI Web of Science (biofilm* or dental plaque or saliva or dental pellicle or mouth or oral cavity) AND (proteomic * or protein*) AND (dental caries). Search was updated on February, 2023. Gray literature was not searched.

### 2.4. Article selection process and eligibility criteria

Observational clinical studies (cross-sectional, longitudinal, or case–control) written in Portuguese, Spanish, English, French, Italian, or German that compared the oral cavity protein abundance (in terms of concentration, activity or levels of total proteins, enzymes, immunoglobulins, antioxidants, and antimicrobial peptides) among caries-affected and caries-free individuals were included. Descriptive or systematic literature reviews, case reports, case series, opinion articles, and letters to the editor as well as studies that evaluated specific s-IgA (against specific microorganisms, for example, *Streptococcus mutans* and *Lactobacillus* spp., among others) and studies that evaluated non-humoral immune response were excluded. The identified records were imported into a reference manager software (EndNoteX9), and duplicate titles were excluded.

All electronically identified records were scanned by title and abstract by two independent researchers (LP and SP). Eligibility of the selected studies was determined by reading the title and abstracts of the articles identified from the electronic databases. Disagreements in this selection process were resolved by a third researcher (ES). Potentially eligible studies were then retrieved for full-text reading. In order to verify reproducibility between reviewers, Cohen's kappa (*k*) was calculated for title (*k* = 0.85) and for abstract (*k* = 0.95) selections. The searches were complemented by manually screening the reference list of the included studies, and those that met the eligibility criteria were also included in this systematic review.

### 2.5. Data extraction

Data such as authors, year of publication, country, study design, number of caries-free and caries-affected participants, mean age of participants, criteria and threshold used for caries diagnosis (cavitated/non-cavitated carious lesions), dental caries experience, type of clinical sample (saliva, pellicle, or dental plaque), sample collection protocol, methods used for protein analysis, and the outcome of interest (concentration, activity or levels of total proteins, immunoglobulins, enzymes, antioxidants, and antimicrobial peptides) were independently extracted by two reviewers (LP and SP) and compiled in an Excel spreadsheet. Disagreements were resolved by a third researcher (ES). Cohen's kappa for data extraction was *k *=* *0.95. In case of incomplete reported data, or full-text was not available, authors were contacted by email. If authors failed to provide additional data or to provide the full-text after two reminders, only published available data were used.

### 2.6. Assessing the methodological quality of individual studies

The methodological quality of individual studies was assessed using the NIH Quality Assessment Tool for Observational Cohort and Cross-Sectional Studies (https://www.nhlbi.nih.gov/health-topics/study-quality-assessment-tools). In addition to the guiding questions, the following question was also assessed: Was the sample collection process clearly described? Each of the guiding questions was classified as “yes” (present) or “no” (absent). For those that were not reported, “NR” was recorded. For final quality estimation purposes, “NR” records were also considered as “no.” Quality was classified as “good” when studies had at least 80% of positive scores, “fair” when it was between 40% and 80%, and “poor” when positive scores corresponded to less than 40% of the criteria (40). The quality assessment was also performed independently by two reviewers (LP and SP) and disagreements were resolved by a third reviewer (ES). Cohen's kappa for this step was *k *=* *0.90.

### 2.7. Synthesis of evidence

Qualitative and descriptive synthesis was performed for all included studies. Quantitative synthesis (meta-analysis) was performed for studies that presented data of the concentration of proteins, immunoglobulins, antioxidants, antimicrobial peptides and activity of enzymes (mean and standard error or standard deviation). Data from these studies were grouped and categorized into “Caries-free” or “Dental Caries.” The meta-analysis was performed using the Review Manager software (RevMan Web, The Cochrane Collaboration 2022) (https://revman.cochrane.org/#/myReviews). Since the primary studies used different methods for assessing the outcomes, the standardized mean difference (SMD) was chosen as a summary statistic in meta-analysis (Cochrane Handbook version 6.4; https://training.cochrane.org/handbook/current). SMD [followed by the respective confidence intervals (CIs)] was calculated for the following outcomes: total protein, immunoglobulins (s-IgA and IgG), carbonic anhydrase VI (CA-VI) and antimicrobial peptides (AMP) (LL-37, hBD, and hNP) concentrations, total antioxidant capacity (TAC) and salivary alpha-amylase, CA-VI, urease, and arginine deaminase system (ADS) activities. Concentrations were converted to mg/dl (for total protein and s-IgA), mmol/L (for total antioxidant capacity), μg/ml (for hBD-2 and hNP), ng/ml (for LL-37), and ng/μl (for carbonic anhydrase). For cohort studies, only data related to the baseline or to follow-up periods were used in the meta-analysis. For studies that dichotomized the results according to participants’ caries risk or experience (low, moderate, or high risk), an average was calculated (considering the number of participants and the standard deviation of each group) as representative of the participants with dental caries (Cochrane Handbook version 6.4). A random-effects model was used with the Inverse Variance statistical method; Cochran's *Q*-test (*χ*^2^) and inconsistency test (*I*^2^) indicated statistical heterogeneity when *p* < 0.10 and values equal to or greater than 50% were obtained, respectively.

## 3. Results

### 3.1. Selection of studies

**Figure 1:**
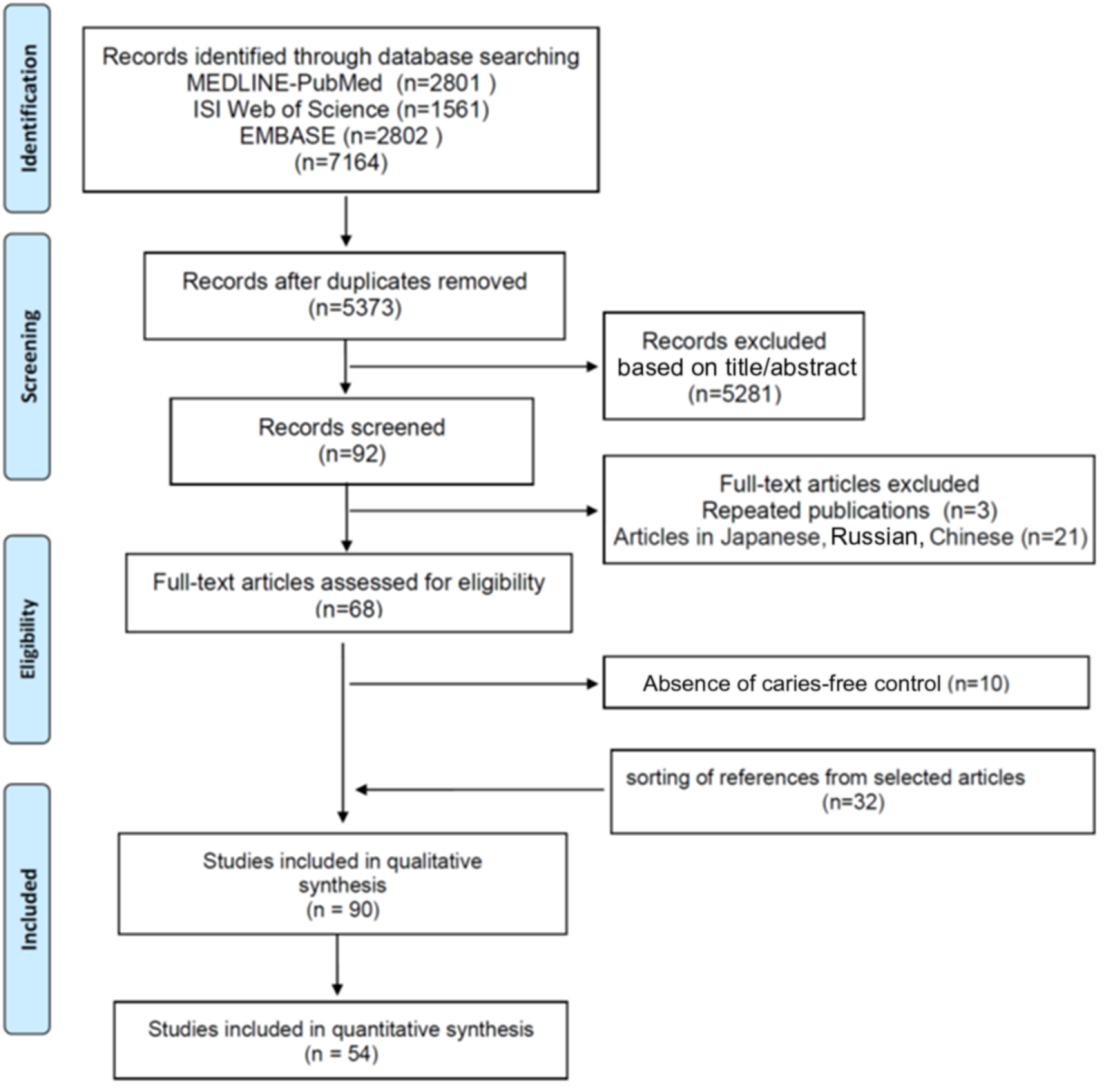
Flow diagram of screened, eligible, and included studies according to PRISMA guideline.

Figure 1 shows the flow diagram of screened, included, and excluded studies. From 68 studies selected for full reading, 10 were excluded because the caries-free group was missing (Supplementary Table S1). Thirty-two studies were manually selected from the reference list of the remaining eligible studies resulting in 90 studies included in the systematic review.

### 3.2. Characteristics of the included studies

Most of the included studies were conducted in Asia (*n *=* *47; 52.2%), followed by America (*n *=* *34; 37.7%) and Europe (*n *=* *9; 10.1%). Only two studies presented a cohort design, being the majority of the cross-sectional studies (*n *=* *88). A total of 6,814 participants were evaluated, with 2,996 being caries-free, and the remaining 3,818 participants were caries-affected ones. Children and adolescents comprised the studied population of most of the studies (*n *=* *67; 74.5%).

Unstimulated saliva was collected in 67 studies (74.5%), stimulated saliva was collected in 15 studies (16.6%), oral rinse or dental plaque were collected in one study each (2.2%), and both dental plaque and unstimulated or stimulated saliva were collected in five studies (5.5%). It was not clear to estimate whether unstimulated or stimulated saliva was collected in one study (1.1%).

Dental caries experience was determined based on the International Caries Detection and Assessment System (ICDAS) (five studies; 5.5%) (22, 31, 34, 41, 42). Twelve studies (13, 21, 43–52) did not describe how caries experience was determined. With the exception of four studies (12, 19, 53, 54) that used other indexes, the remaining studies used the DMF index to assess dental caries experience (69 studies; 76.7%) (11, 14–18, 20, 23–28, 30, 33, 36, 55–107). In all studies, caries-free individuals presented dmft/DMFT or dmfs/DMFS equal to zero, while caries-affected ones presented dmfs/DMFS from 1 to 48 and dmft/DMFT from 1 to 30. Regarding the threshold for dental caries detection, most of the studies (60 studies; 66.7%) (11, 14, 15, 20, 23–28, 33, 37, 55–60, 62–78, 81, 83–97, 99–106) used the WHO criteria (e.g., frank dentinal cavitation). Initial non-cavitated lesions were also considered in nine studies (10%) (16, 18, 31, 36, 61, 79, 80, 82, 98).

### 3.3. Qualitative synthesis

Twenty-one studies reported salivary total protein concentration as outcome (11–13, 16–17, 20, 24, 25, 28, 46, 52, 54, 56, 58, 63, 72, 75, 79, 85, 90, 96) (Supplementary Table S2). Biuret reaction, Bradford, and Lowry were the most used methods for the outcome quantification (76.2%). Ten studies (47.6%) showed higher total protein concentration in the oral cavity of caries-affected individuals (47.6%) (11–13, 28, 72, 75, 78, 85, 90, 96), whereas an opposite trend was observed in two studies (17, 52). In the remaining nine studies (43%), no differences were found in the salivary total protein concentration among individuals.

The TAC of saliva was reported in 19 studies (12, 13, 24, 41, 48, 55, 57, 59, 65, 66, 72, 78, 81, 85, 87, 89, 92, 93, 96) (Supplementary Table S3) with ferric-reducing antioxidant power (FRAP) and thiobarbituric acid reactive substances (TBARS) being the methods most used (26% of studies each). Fifteen studies (79%) (12, 13, 41, 48, 55, 59, 65, 66, 72, 78, 81, 85, 87, 93, 96) showed that caries-affected individuals present higher TAC. Higher TAC in caries-free individuals was found in one study (89) and no differences were found among individuals in the other three remaining studies (24, 57, 92).

Twenty-four studies reported s-IgA as outcome (14–17, 27, 31, 43, 52, 61–63, 68, 75, 77, 79, 80, 86, 88, 90, 94, 99, 102, 105, 107) (Supplementary Table S4). Enzyme-linked immunosorbent assay (ELISA) was the method used in most of the studies (41.6%). Eleven studies (46%) showed that the oral cavity of caries-free individuals present higher s-IgA concentration (17, 27, 31, 52, 62, 68, 75, 79, 90, 99, 107). Higher s-IgA concentration was found in the oral cavity of caries-affected individuals by eight studies (33%) (14–16, 43, 63, 88, 102, 105). No difference was observed among caries-free and caries-affected individuals by the remaining studies.

Three studies reported salivary IgG concentration in caries-free and caries-affected individuals (15, 68, 90) (Supplementary Table S4). One study showed higher levels in caries-affected individuals (15), whereas another study (68) showed higher salivary levels in caries-free individuals. Razi et al. (90) did not find any difference among individuals for the IgG levels.

Superoxide dismutase (SOD) activity was reported in three studies (12, 69, 96). All three studies showed that SOD activity was higher in caries-affected individuals. Myeloperoxidase activity was higher in caries-affected individuals (67). Salivary glutathione peroxidase activity was reported in two studies (67, 70) with conflicting results. Lactoperoxidase activity and hypothiocyanite and thiocyanate concentrations were similar among caries-free and caries-affected individuals (73) (Supplementary Table S5).

Eight studies reported activity or concentration of salivary alpha-amylase (19, 47, 54, 58, 63, 84, 97, 107) (Supplementary Table S6). Balekjian et al. (58) and Singh et al. (97) showed higher alpha-amylase concentration and activity, respectively, in caries-affected individuals. Mojarad et al. (47), Borghi et al. (19), and Ahmad et al. (107) showed lower alpha-amylase activity in caries-affected individuals. In three studies (54, 63, 84), no differences were observed on the activity or concentration among individuals.

Lysozyme concentration or activity was reported in four studies (20, 30, 49, 64) (Supplementary Table S7). Higher activity was found in caries-affected individuals (20), whereas lower lysozyme concentration was found in those individuals (30). In the other two studies, no differences were found among individuals. Two studies reported salivary lactoferrin concentration (30, 64) (Supplementary Table S7). No differences were observed among the studied individuals. Yang et al. (106) showed higher proteinase-3 concentration in caries-free individuals. Hedenbjörk-Lager et al. (11) showed higher concentration of metalloproteinase-8 in caries-affected individuals (Supplementary Table S7).

Activity or concentration of CA-VI was reported in seven studies (18, 19, 26, 50, 82, 83, 98) (Supplementary Table S8). Four studies (19, 82, 83, 98) showed higher CA-VI activity in saliva and in dental plaque of caries-affected individuals. CA-VI concentration was higher in saliva or in dental plaque of caries-free individuals (82, 83). The other two studies did not find differences among individuals.

Urease activity, reported as ammonia production from urea, was shown by four studies (74, 76, 91, 95) (Supplementary Table S9). Lower urease activity was found in the dental plaque (76, 91, 95) and in the saliva of caries-affected individuals (74, 91). Three studies reported ADS activity in the oral cavity as ammonia produced from arginine breakdown (42, 74, 91), and one study reported ADS activity as citrulline produced from arginine (42) (Supplementary Table S9). Lower ADS activity was found in saliva (76, 91) and in the dental plaque (91) of caries-affected individuals. However, similar ADS activity was found in saliva (42, 74) and in the dental plaque (42, 76) of caries-free and caries-affected individuals. One study (104) showed arginine and lysine levels to be higher in the saliva of caries-free individuals.

Higher albumin and cystatin-S concentrations were found in the oral cavity of caries-free individuals (36, 52). Two studies reported mucin concentration, with higher MUC5B and MUC7 in caries-free (33) or higher mucin concentration in caries-affected individuals (17). Histatin concentration was not different among caries-free and caries-affected individuals (54), while another study reported that histatin was more likely to be non-detectable or being in reduced concentration in caries-free individuals (51). One study found higher proline-rich protein (PRPs) concentration in caries-affected individuals (100), whereas no difference among caries-free and caries-affected individuals was found for acidic and basic PRPs (54). One study reported a greater number of PRP bands on sodium dodecyl-sulfate polyacrylamide gel electrophoresis (SDS-PAGE) from saliva of caries-free individuals (60). Electrophoresis-based analysis showed higher levels of positive-charged proteins on the saliva of caries-affected individuals (58). Moreover, protein bands of 15 kDa (suspected as cystatin), 25 kDa (suspected as basic PRPs), 60 kDa (suspected as alpha-amylase), 65 kDa (suspected as serum albumin), and 95 kDa (suspected as the secretory component of IgA) were more frequently found in caries-free individuals (44), whereas 17 kDa protein band was more frequently found in caries-active ones (25) (Supplementary Table S10).

AMP concentration in the oral cavity was assessed in nine studies (21–23, 28, 34, 45, 53, 101, 103), with conflicting results (Supplementary Table S11). One study showed lower LL-37 concentration in the saliva of high-caries activity individuals (23), whereas similar LL-37 concentration was found in the saliva of the individuals irrespective of caries status in other studies (28, 34, 53). In one study, hBD concentration was higher in caries-free individuals (34) but other two studies showed higher concentration of hBD-2 and hBD-4 in caries-affected individuals (21, 22). The other two studies (28, 53) did not find differences among caries-free and caries-affected individuals. One study reporter higher levels of hNP1–3 in the saliva of caries-free individuals (101), and another study showed higher levels in the oral cavity of caries-affected individuals (45). Three studies found no differences on hNP1–3 or hNP-4 levels among caries-free and caries-affected individuals (21, 28, 103).

### 3.4. Quantitative synthesis

**Figure 2:**
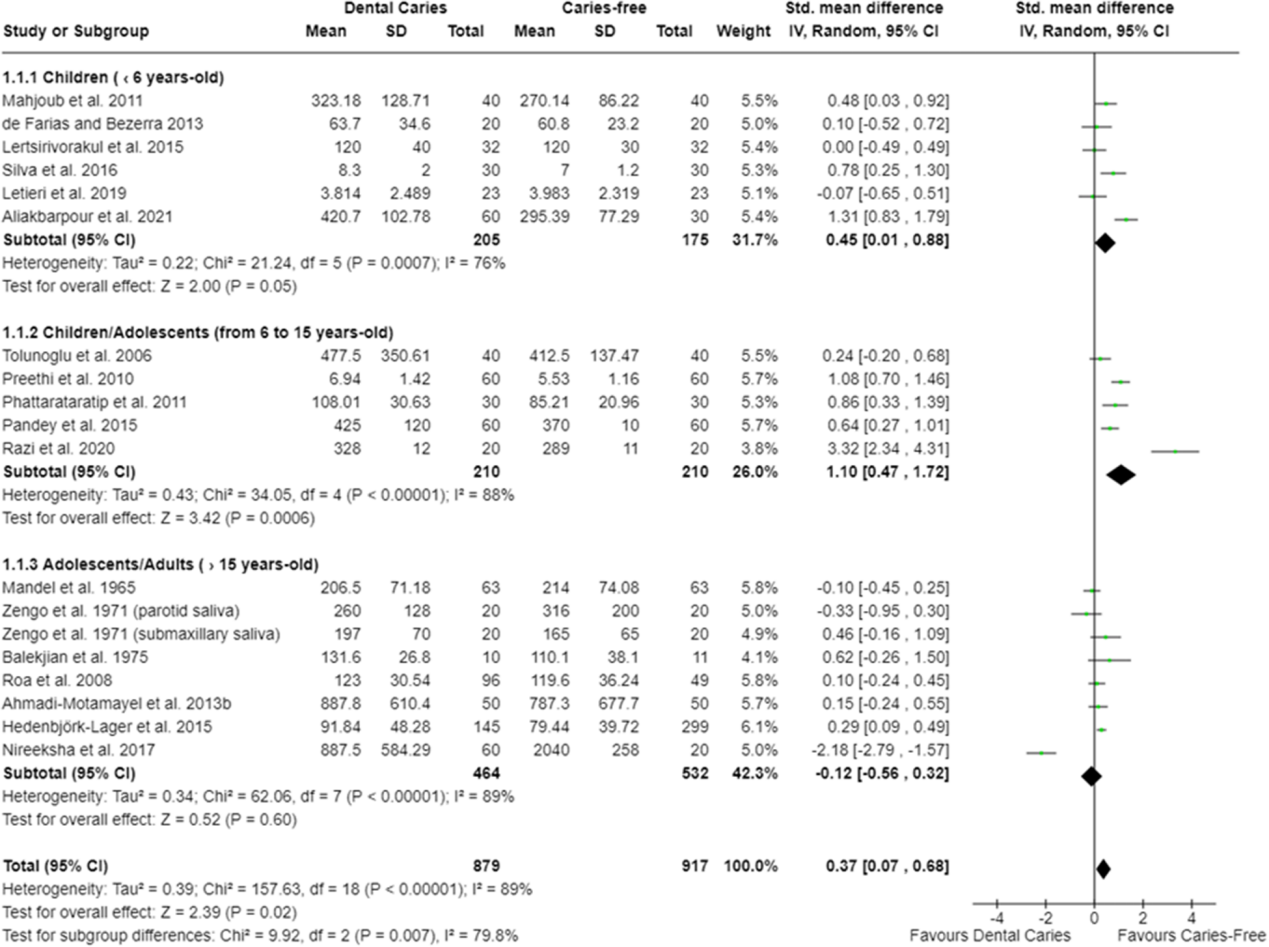
Standardized mean difference (SMD) and confidence intervals for total protein concentration (mg/dl) in the oral cavity of caries-free and caries-affected individuals. Positive SMD values mean lower total protein concentration in caries-free individuals (favours caries-free). Negative SMD values mean lower total protein concentration in caries-affected individuals (favours dental caries).

**Figure 3:**
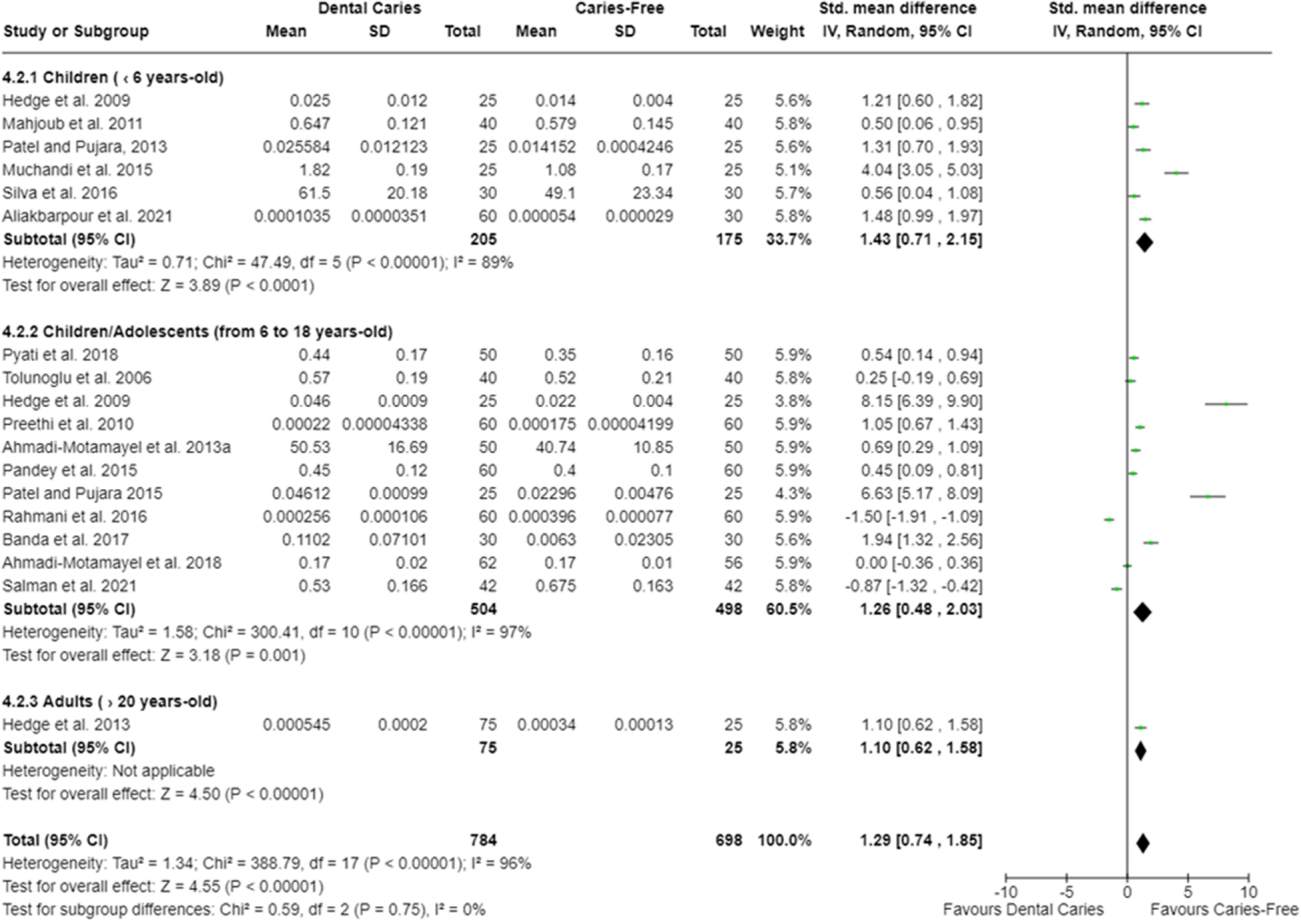
Standardized mean difference (SMD) and confidence intervals for TAC (mmol/L) in the oral cavity of caries-free and caries-affected individuals. Positive SMD values mean lower TAC in caries-free individuals (favours caries-free). Negative SMD values mean lower TAC in caries-affected individuals (favours dental caries).

Twelve meta-analyses were performed. The oral cavity of caries-free children (up to 6 years old) [SMD = 0.45 (95% CI: 0.01–0.88); *p* = 0.05] and of caries-free children/adolescents (from 6 to 15 years old) [SMD = 1.10 (95% CI: 0.47–1.72); *p* = 0.0006] presented lower total protein concentration (18 studies) compared with caries-affected ones. Moreover, irrespective of the participant age, caries-free individuals presented lower salivary total protein concentration [SMD = 0.37 (95% CI: 0.07–0.68); *p* = 0.02] (Figure 2). Studies also showed that caries-free individuals had lower TAC levels [SMD = 1.29 (95% CI: 0.74–1.85); *p* < 0.00001; 17 studies] (Figure 3), being this trend observed in children, adolescents, and adults.

**Figure 4:**
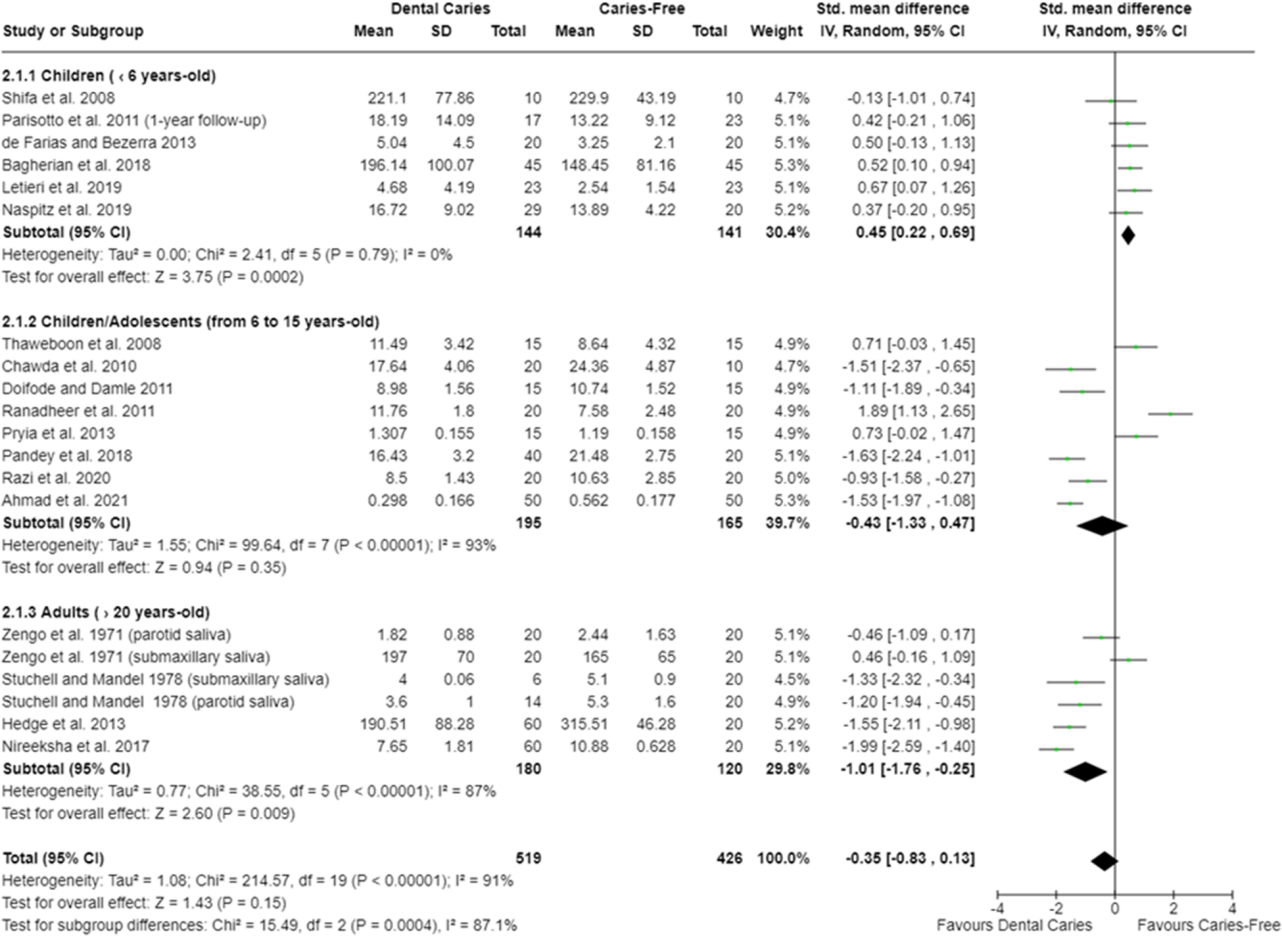
Standardized mean difference (SMD) and confidence intervals for s-IgA concentration (mg/dl) in the oral cavity of caries-free and caries-affected individuals. Positive SMD values mean lower s-IgA concentration in caries-free individuals (favours caries-free). Negative SMD values mean lower s-IgA concentration in caries-affected individuals (favours dental caries).

For s-IgA, the oral cavity of caries-free children (up to 6 years old) had lower concentration [SMD = 0.45 (95% CI: 0.22–0.69); *p* = 0.0002], whereas lower s-IgA concentration was found in the oral cavity of caries-affected adults [SMD = −1.01 (95% CI: −1.76 to −0.25); *p* = 0.009]. Irrespective of the age of the participants, the oral cavity of caries-free and caries-affected individuals harbored similar s-IgA concentration [SMD = −0.35 (95% CI: −0.83 to 0.13); *p* = 0.15; 18 studies] (Figure 4). In addition, no difference on salivary IgG levels among caries-free and caries-affected individuals were observed [SMD = −0.12 (95% CI: −0.75 to 0.51); *p* = 0.71; three studies] (Supplementary Figure S1).

**Figure 5:**
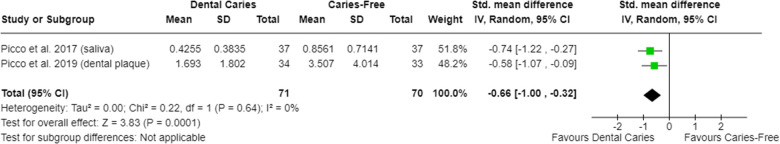
Standardized mean difference (SMD) and confidence intervals for carbonic anhydrase VI concentration (CA-VI; ng/μl) in the oral cavity of caries-free and caries-affected individuals. Negative SMD values mean lower CA-VI concentration in caries-affected individuals (favours dental caries).

**Figure 6:**
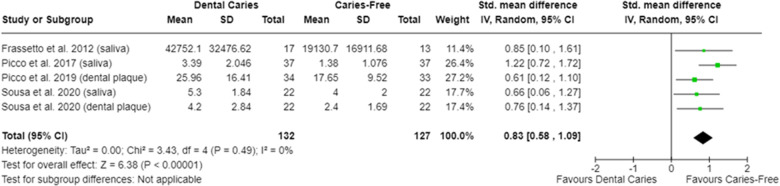
Standardized mean difference (SMD) and confidence intervals for carbonic anhydrase VI activity levels (CA-VI; pixel area or pixel area/mg protein) in the oral cavity of caries-free and caries-affected individuals. Positive SMD values mean lower CA-VI activity in caries-free individuals (favours caries-free).

**Figure 7:**
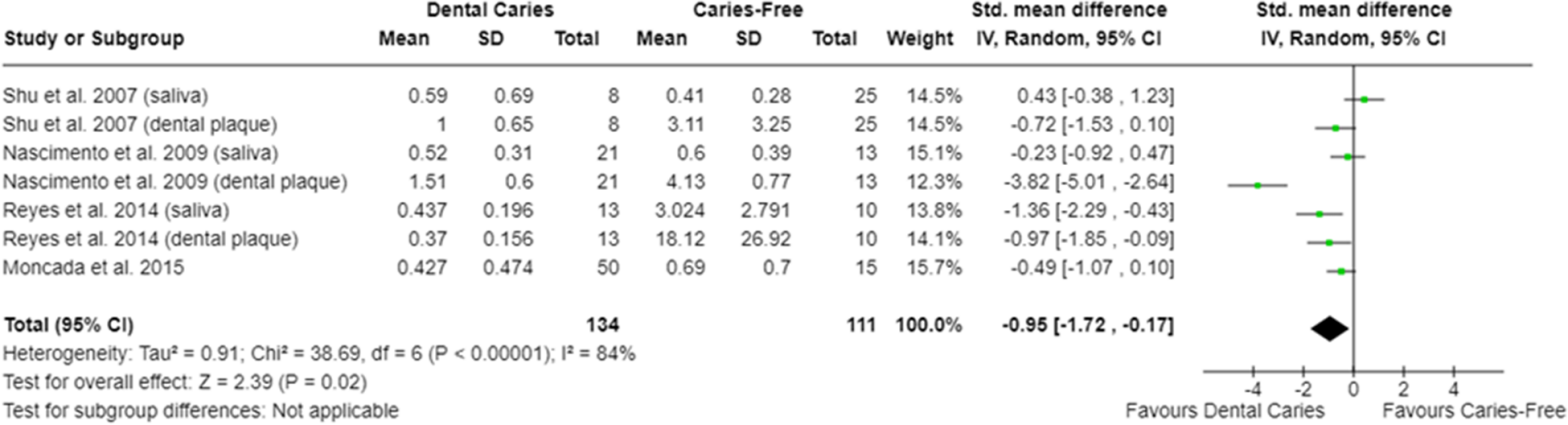
Standardized mean difference (SMD) and confidence intervals for urease activity levels (μmol ammonia/min/mg protein) in the oral cavity of caries-free and caries-affected individuals. Positive SMD values mean lower urease activity levels in caries-free individuals (favours caries-free). Negative SMD values mean lower urease activity levels in caries-affected individuals (favours dental caries).

**Figure 8:**
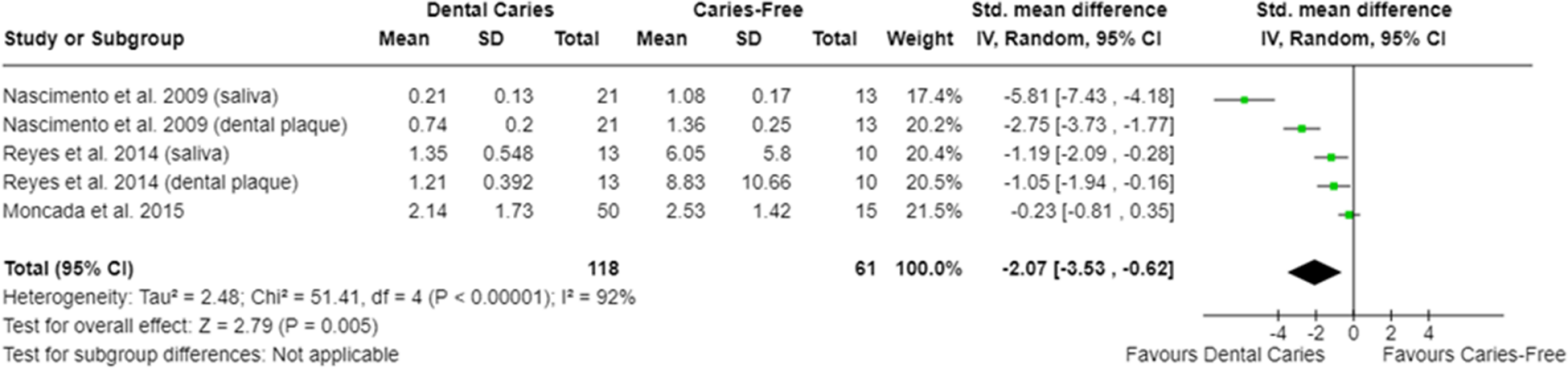
Standardized mean difference (SMD) and confidence intervals for ADS activity levels (μmol ammonia/min/mg protein) in the oral cavity of caries-free and caries-affected individuals. Negative SMD values mean lower ADS activity in caries-affected individuals (favours dental caries).

In relation to salivary enzymes, alpha-amylase activity was not different among caries-free and caries-affected individuals [SMD = −0.36 (95% CI: −0.84 to 0.11); *p* = 0.13; four studies] **(**Supplementary Figure S2**)**. The oral cavity of caries-affected individuals had lower CA-VI concentration [SMD = −0.66 (95% CI: −1.00 to −0.32); *p* = 0.0001] (Figure 5), whereas the oral cavity of caries-free individuals had lower CA-VI activity [SMD = 0.83 (95% CI: 0.58 to 1.09); *p* < 0.00001; three studies] (Figure 6). Caries-affected individuals had lower urease activity [SMD = −0.95 (95% CI: −1.72 to −0.17); *p* = 0.02; four studies] (Figure 7) and lower ADS activity [SMD = −2.07 (95% CI: −3.53 to −0.62); *p* = 0.005; three studies] (Figure 8). The meta-analysis of AMP showed no differences on LL-37, hBD, and on hNP concentrations among caries-free and caries-affected individuals (Supplementary Figures S3–S5).

### 3.5. Methodological quality of individual studies

Among the cross-sectional studies, the methodological quality of 53 studies (60.2%) was classified as “fair,” 29 studies (33%) were classified as “good,” and six studies (6.8%) were classified as “poor.” The guiding questions that received a negative answer (no) or that the authors did not report (NR) were Q8 (*n* = 88 studies), Q4 (*n* = 75 studies), Q10 (*n* = 43 studies), Q6 (*n* = 29 studies), Q2 (*n* = 25 studies), Q3 (*n* = 15 studies), Q1 (*n* = 4 studies), and Q5 (*n* = 1 study). Among the cohort studies, the quality of evidence of one was classified as “fair” and the other study was classified as “good.” The guiding questions that received a negative answer (no) or that the authors did not report (NR) were Q2 (*n* = 2 studies), Q5 (*n* = 2 studies), Q12 (*n* = 2 studies), Q4 (*n* = 1 study), Q6 (*n* = 1 study), Q9 (*n* = 1 study), and Q15 (*n* = 1 study) (Supplementary Tables S12, S13).

## 4. Discussion

The results of this systematic review and some of the meta-analyses indicated that there are some proteins in the oral cavity that could potentially be used for the identification of caries-free or caries-affected individuals.

As observed in the meta-analysis, the saliva of caries-free individuals had lower total protein concentration in comparison with caries-affected individuals (Figure 2). It has been reported that the salivary total protein concentration tends to increase according to carious lesions extension (i.e., the presence of cavitated lesions) as a result of dentinal organic matrix degradation by MMPs activity (12). In fact, higher levels of matrix metalloproteinases (MMP), specifically MMP-8, have been identified in the saliva of caries-affected individuals (11) (Supplementary Table S7). Furthermore, it is considered that the higher protein concentration in the saliva of caries-affected individuals may be a protective and/or an adaptive response of the body against dental caries, considering the important antimicrobial properties performed by saliva (96). Some authors suggest that this increase in total protein concentration would also be a consequence of an increase in salivary alpha-amylase levels in caries-affected individuals (58, 75).

Some studies included in this systematic review indicated that caries-affected individuals had a high concentration or activity of salivary alpha-amylase (58, 97) (Supplementary Table S5). This enzyme has the ability to act as a component of the acquired salivary pellicle, providing binding sites for oral microorganisms (108, 109). Salivary amylase can also bind to the surface of certain microorganisms, such as *Actinomyces* spp*.* and *Streptococcus* spp., including *Streptococcus mutans* (110). In both cases, besides aiding microbial adhesion, this enzyme promotes starch breakdown leading to shorter glucose-containing polymers that can be either converted into acids by microorganisms or act as precursors for the synthesis of insoluble extracellular polysaccharides by *S. mutans* (111). Therefore, it is understood that the greater the salivary alpha-amylase activity, the greater the potential for the development of a highly cariogenic dental plaque. However, the results of the other studies included in this systematic review are contradictory, and the meta-analysis showed no difference in alpha-amylase activity among caries-free and caries-affected individuals (Supplementary Figure S2). It is possible that methodological differences among studies have contributed to these discrepant results and further studies are needed to better clarify whether an association between salivary amylase activity and dental caries exists.

The meta-analysis indicated that caries-free individuals have lower TAC levels compared with caries-affected ones (Figure 3). This result agrees with previous studies that showed high TAC levels in caries-affected individuals (12, 13, 41, 48, 55, 59, 65, 66, 72, 78, 81, 85, 87, 93, 96). It is well-known that reactive oxygen species (ROS) cause DNA and protein damage, lipid peroxidation, and stimulation of proinflammatory cytokine secretion (41, 59, 78, 81, 87, 93). Oxidative stress occurs as a result of the imbalance among free radicals (FR), ROS, and compounds with antioxidant activity (65). It has been discussed that the increase in TAC occurs as a compensatory event to neutralize the oxidative effects induced by FR and ROS produced by both microorganisms and by the host during the disease development (55, 78, 96). This increase in TAC levels could even enable the growth of cariogenic microorganisms, since the more antioxidants are produced, the more reactive molecules (FR and ROS) are neutralized, negatively affecting the antimicrobial effects of these molecules on the oral microorganisms (41). Furthermore, a positive correlation between greater total protein concentration in saliva and higher TAC in caries-affected individuals has been reported (72, 78, 84, 85, 96). It is argued that this increase on TAC levels in caries-affected individuals might also occur due to an increase in salivary peroxide levels (65, 66, 85, 87, 93, 96), which constitute one of the main antioxidant systems found in the oral cavity. Some studies indicated that the activity of the antioxidant enzymes, as SOD, myeloperoxidase, and glutathione peroxidase, is higher in caries-affected individuals (12, 67, 69, 96) (Supplementary Table S5). SOD and myeloperoxidase exert an antimicrobial effect (112), while the glutathione peroxidase, in turn, catalyzes the reduction of hydroperoxides, including hydrogen peroxides, into reduced glutathione, protecting cells from the oxidative stress (112). Thus, it is likely that several antioxidant systems are more expressed in the oral cavity of caries-affected individuals.

In relation to carbonic anhydrase, the meta-analysis showed that caries-affected individuals have lower CA-VI levels and caries-free individuals present lower CA-VI activity, both in saliva and in dental plaque (Figures 5, 6). This agrees with the results reported by Wang et al. (113, 114) who showed a greater enrichment of metabolic pathways associated with CA-VI in the saliva of caries-affected individuals (114) being this suggestive of higher CA-VI activity. It is discussed that pH fluctuations are quickly buffered in the oral cavity of caries-free individuals since these individuals present higher CA-VI salivary concentration (82, 83). However, as a consequence of its lower levels in caries-affected individuals, CA-VI is being activated more constantly in those individuals in an effort to control the acidification promoted by the metabolic activity of microorganisms. Conversely, it may explain the lower CA-VI activity in caries-free individuals. It is important to mention that the activity of CA-VI, both in saliva and in the dental plaque, was able to adequately discriminate caries-affected (presenting non-cavitated lesions) of caries-free individuals (98), indicating that this parameter can potentially be used to identify individuals at greater risk for dental caries development.

Regarding the synthesis of alkaline compounds, caries-affected individuals have lower urease (Figure 7) and lower ADS (Figure 8) activities compared with caries-free ones, which agrees with previous evidence (42, 74, 76, 91, 95, 115). The production of ammonia from urea and from arginine metabolism has been considered one of the protective mechanisms against the harmful effects of oral acidic environments. The greater activity of ADS would be compatible with the high levels of arginine in the oral cavity of caries-free individuals (104). It is understood that the differences observed among caries-free and caries-affected individuals in the ability of the oral microbiota to produce ammonia (from arginine or urea) are due to the fact that alkali-generating enzymes are more abundant in the oral cavity of caries-free individuals (76, 91). This may be related to the fact that there is a greater abundance of alkali-producing microorganisms (as *Streptococcus sanguinis* and *Streptococcus gordonii*) in the oral cavity of caries-free individuals (91).

Other salivary enzymes also appear to be differentially abundant among caries-free and caries-affected individuals. This is the case, for example, of protease-3 (PR3), which is present at low concentrations in the oral cavity of caries-affected individuals (106). Studies reported that the ideal condition for PR3 activity is at pH around 8.0 (116, 117). As the pH of the oral cavity of caries-free individuals tends to be more alkaline than that of caries-affected ones (118), this would help explain the low levels of PR3 in caries-affected individuals (Supplementary Table S7). In addition, PR3 exerts an antimicrobial effect (119) and it participates in the synthesis of antimicrobial peptides, specifically LL-37 (120). This could explain the low levels of this protease in individuals with caries, although the exact role played by this protease in the oral cavity is still unknown. Regarding lysozyme and lactoferrin, the results are conflicting (20, 30, 49, 58, 64) or no differences were observed among the studied individuals (30, 64) (Supplementary Table S7). The studies included in this review do not allow us to conclude whether there is, in fact, any association between salivary levels and/or activities of lysozyme, lactoferrin, and PR3 with oral health or with dental caries.

In three studies included in this review, cystatin and albumin concentrations were higher in the oral cavity of caries-free individuals (36, 44, 52) (Supplementary Table S10). This result agrees with other studies showing higher cystatin concentration in the saliva or in the dental plaque of caries-free individuals (114, 121, 122) This association is plausible since cystatin exerts an antimicrobial effect by acting as a proteinase inhibitor (cysteine proteinase type) (123, 124), and it also contributes to the maintenance of calcium supersaturation onto the tooth surface, a function also played by PRPs, histatins, and statherins (125). Albumin may help on acid buffering (126) thus playing an important role in the oral cavity. Furthermore, similar levels of statherins and agglutinins are found in the oral cavity of caries-free and of caries-affected individuals, this finding being reported by only one study included in this systematic review (51, 54) (Supplementary Table S10). However, conflicting results were observed in relation to the concentration of mucin, PRPs, and histatin (33, 44, 51, 54, 60) among caries-free and caries-affected individuals (Supplementary Table S10). MUC5B has a high affinity for hydroxyapatite, which tends to inhibit microbial adhesion onto the tooth surface (127–130). On the other hand, MUC7 remains free in the salivary aqueous phase, and it exerts an antimicrobial effect by interacting with bacterial cell surface (131). This would explain their greater concentration in individuals without caries (33). Despite these findings, an opposite trend (i.e., higher mucin concentration in the oral cavity of caries-affected individuals) was also observed (17). Agglutinin and statherin concentrations seemed to be similar among caries-free and caries-affected individuals (51, 54).

The meta-analysis showed that caries-free children (up to 6 years old) have lower s-IgA levels (Figure 4). In sharp contrast, Al Amoudi et al. (14) showed higher s-IgA in caries-affected children that is attributed to the increase in the antigenic load promoted by cariogenic microorganisms, which leads to a greater production of antibodies. Considering that the child's immune system is still under maturation and under development, exposure to antigens from a cariogenic microbiota may be stimulating sensitization and antibody production, contributing to the higher levels of s-IgA in this group of individuals. Furthermore, other authors also argued that s-IgA levels are positively associated with increased counts of *S. mutans* in the oral cavity of caries-affected individuals (15, 16, 63, 88, 102, 105). Although this is a plausible explanation due to the importance played by the *S. mutans* in the cariogenic dental plaque, it should be noted that the clinical significance of these associations is limited. Actually, the composition of dental plaque microbiome is much more diverse, being dependent on caries activity and exhibiting differences among individuals (132). Therefore, the statement that s-IgA concentrations are directly related to the levels of certain microorganisms does not seem logical within an ecologically broader concept of dental caries as a dysbiosis-induced disease. Furthermore, if this association was always true, caries-affected adults would also present higher levels of s-IgA, which is in disagreement with the results presented in our meta-analysis (Figure 4) and also in relation to the results reported by other studies (17, 31, 68) (Supplementary Table S4). We know that s-IgA levels depend on the host’s age. In general, adults have higher s-IgA levels compared with children (133). Despite this increased level, and considering that s-IgA not only inhibits the microbial adhesion to dental surfaces but also promotes microbial agglutination and acts synergistically in relation to important salivary enzymes, such as lactoferrin and peroxides (134), it is understood that a reduction in s-IgA levels places individuals at greater risk for caries development. However, irrespective to the participants’ age, no clear pattern exists on s-IgA levels among caries-free and caries-affected individuals.

Some authors suggested that a greater expression of AMPs in the saliva of caries-affected individuals may be due to both the simultaneous presence of gingival inflammation (28, 103) and the high salivary levels of *S. mutans* (53). However, it is still discussed that, despite exerting an antimicrobial effect, the higher AMP levels in caries-affected individuals mean that the virulence of the microorganisms present in the oral cavity of these individuals (specifically *S. mutans*) is higher when compared to caries-free individuals, overcoming any antimicrobial effect played by these AMPs (28, 45). It is also considered that higher salivary levels of beta-defensins in caries-free individuals are associated with higher salivary levels of the amino-acids lysine and arginine (135), since both are components of these AMPs. Our meta-analysis, however, did not show differences on the salivary levels of LL-37, hBD, and hNP among caries-free and caries-affected individuals (Supplementary Figures S3–S5). Considering each study individually, the reported outcomes are contradictory (21, 22, 23, 28, 34, 45, 53, 101, 103) (Supplementary Table S11). In general, an expressive variability in the AMP concentration among different studies was observed. It is speculated that this variability may occur due to polymorphisms, due to the number of codifying AMP genes among the evaluated individuals (34, 101), or due to the fact that immune response maturates with aging, and, therefore, lower salivary levels of AMP could be found in children (23, 34, 101, 103).

The composition of saliva and its biological characteristics are influenced by several factors, such as participants’ age, salivary flow, type of salivary stimulus, type of salivary gland, diet, degree of hydration, circadian cycle, among others (136–138). Thus, all these factors must be adequately controlled and adjusted in order to minimize potential sources of variability and bias in the analyses. We observed that most of the included studies lacked a proper control of confounder for salivary flow, for participant’s age and diet or for degree of hydration. Therefore, it is possible that these uncontrolled factors could have been the source of the variability observed in some of the outcomes analyzed in this review. Another important source of variation is the origin (or source) of saliva. While total whole saliva has a composition that is representative of the fluids secreted by all major and minor salivary glands as well as by gingival crevicular fluid, saliva collected directly from a specific gland has a protein content that can be distinct when compared to whole saliva. In addition to these factors, the presence or absence of stimuli during saliva collection can also affect salivary composition. This occurs due to the participation of different glands in stimulated and non-stimulated salivary flow. As can be seen in the Supplementary Tables presented in this review, saliva collection procedures were highly variable among studies. Different proteomic profiles can be obtained from these different analyzes as previously observed by other authors (135, 139). An important point to be emphasized is the influence of the circadian rhythm on saliva composition. Ideally, studies should collect samples at the same time of day (136, 140). Some studies reported saliva collection in the morning between 8 a.m. and 11 a.m., before eating, drinking, and before performing oral hygiene. This is probably the best method to avoid contamination of saliva with external factors, minimizing any possible effect or influence of the diet on saliva composition (37).

Many studies reported the caries experience of the participants through the dmfs/dmft or DMFS/DMFT indexes. The threshold for carious lesions detection was set as the presence of frank cavitation (following the WHO criteria). In many cases, the decayed component of DMF index was not individually reported. Only few studies reported caries experience at early-lesion threshold. Moreover, caries activity at the lesion level was not assessed in most of the studies. Ideally, both the earliest stages of carious lesions (when a cavitation is clinically absent) and the assessment of the activity of the carious lesion should be simultaneously considered. This will allow strategies to be implemented immediately and at an early moment, enabling disease control at the individual level.

It is also important to highlight the high heterogeneity found in the meta-analyses. This indicates a high variability among studies likely attributed to methodological differences (i.e., collection of saliva or dental plaque; distinct methodologies for the reported outcomes), which may affect the magnitude and direction of the observed effects. Moreover, the statistical heterogeneity indicates that a common effect is not observed among all studies being this effect also dependent on the studied participants. Furthermore, heterogeneity estimation of meta-analysis performed with few studies (less than 10 primary studies) needs to be analyzed with care. In order to minimize heterogeneity, subgroup analysis was performed according to participants’ age and the outcomes were converted into the same unit of concentration or activity, and the effect size was tested by a random-effects model. Nevertheless, it is important to acknowledge that the conclusion of this study is based on highly heterogeneous studies. In addition, the small number of studies in most of the meta-analyses is also a concern. We focused our searches on the three main databases (MEDLINE-PubMed, ISI Web of Science, and EMBASE). As other databases were not assessed, one may argue that other relevant studies may not have been identified by the search strategies used in this review and that the inclusion of new studies may change the magnitude and direction of the effects presented in the meta-analyses. While we acknowledge those concerns as limitations of the study, it is important to mention that more than 5,000 studies were retrieved from the above-mentioned databases and a hand-search was also performed in the reference list of the included studies to maximize the screening of the potential eligible studies not identified by the search strategy. Furthermore, the methodological quality of most of the included studies was classified as fair and some important methodological issues were identified, such as lack of sample size justification and study power calculation, absence of a clear description of the blinding process, study participants not clearly defined, as well as a lack of a proper description of the methods and conditions for sample collection. The above-mentioned limitations need to be taken into consideration.

## 5. Conclusion

**Figure 9:**
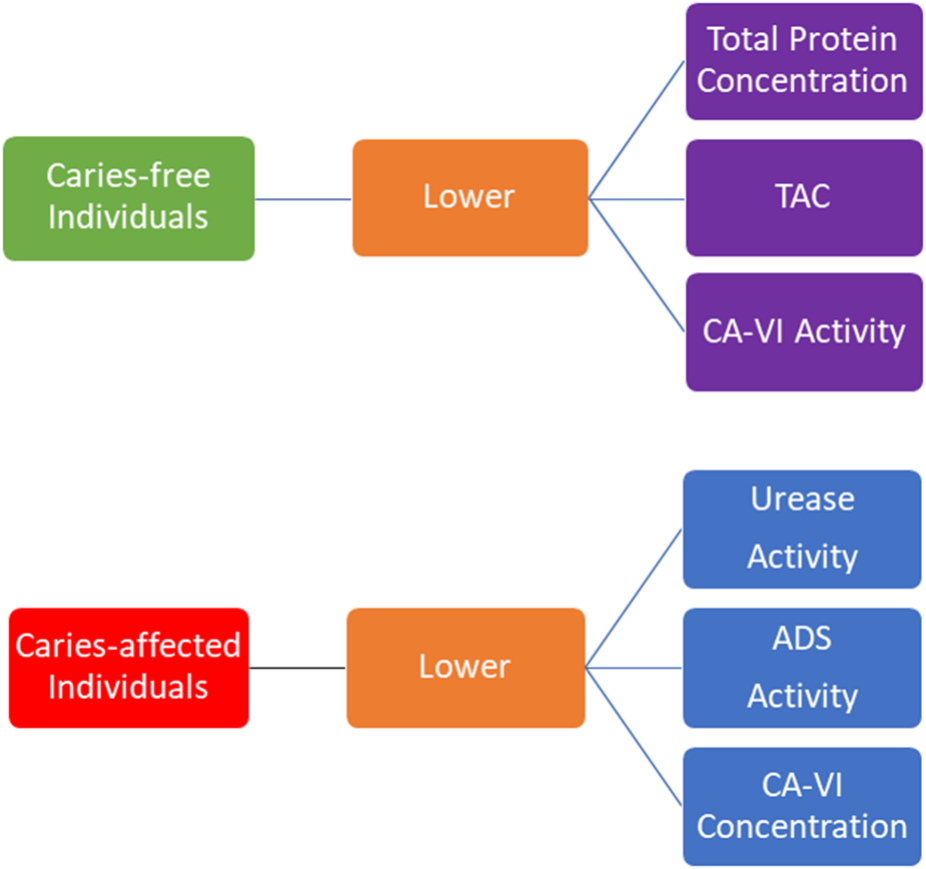
Oral cavity protein abundance among caries-free and caries-affected individuals.

This systematic review and meta-analyses indicated important differences on oral cavity protein abundance among caries-free and caries-affected individuals (Figure 9). Meta-analyses indicated lower total protein concentration, lower TAC, and lower CA-VI activity in the oral cavity of caries-free individuals, whereas lower CA-VI concentration, and lower urease and ADS activities were found in the oral cavity of caries-affected individuals. These differences may indicate potential protein patterns associated with the oral health or with the dental caries conditions identifying individuals who are at greater risk of disease development. However, even when statistically significant, some of the results were not very consistent. In addition, the diagnostic value of these patterns and their validity need to the evaluated by long-term cohort studies in the future.
