# Supplementary material for: Comparison of oral cavity protein abundance among caries-free and caries-affected individuals—a systematic review and meta-analysis
Source: Front Oral Health. 2023 Sep 15;4:1265817. doi: 10.3389/froh.2023.1265817 (PMC10540632; doi:10.3389/froh.2023.1265817)
Supplement: Supplementary file 1 [file Table1.docx]

**Table S1**. Excluded studies and main reason for exclusion.

| **First author, year** | **Reason for exclusion** |
| --- | --- |
| Challacombe; Lehner; Guggenheim, 1972 | Absence of caries-free individuals |
| Dewar; Parfitt, 1954 | Absence of caries-free individuals |
| Gandhy; Damle, 2003 | Absence of caries-free individuals |
| Gornowic *et al*., 2014 | Absence of caries-free individuals |
| Kedjarune *et al*., 1997 | Absence of caries-free individuals |
| Kimoto *et a*l., 2006 | Absence of caries-free individuals |
| Kulhavá *et al*., 2018 | Absence of caries-free individuals |
| Orstavik; Brandtzaeg, 1975 | Absence of caries-free individuals |
| Patil *et al*., 2016 | Absence of caries-free individuals |
| Pekovic; Adamkiewicz; Gornitsky, 1988 | Absence of caries-free individuals |

**References**

1. Challacombe SJ, Lehner T, Guggenheim B. Serum and salivary antibodies to glucosyltransferase in dental caries in man. Nature. 1972 Jul 28;238(5361):219.

2. Dewar MR, Parfitt GJ. Mucin content, physical properties of saliva and caries activity. J Dent Res. 1954 Dec;33(6):751-6.

3. Gandhy M, Damle SG. Relation of salivary inorganic phosphorus and alkaline phosphatase to the dental caries status in children. J Indian Soc Pedod Prev Dent. 2003 Dec;21(4):135-8.

4. Gornowicz A, Tokajuk G, Bielawska A, Maciorkowska E, Jabłoński R, Wójcicka A, Bielawski K. The assessment of sIgA, histatin-5, and lactoperoxidase levels in saliva of adolescents with dental caries. Med Sci Monit. 2014 Jun 29;20:1095-100.

5. Kedjarune U, Migasena P, Changbumrung S, Pongpaew P, Tungtrongchitr R. Flow rate and composition of whole saliva in children from rural and urban Thailand with different caries prevalence and dietary intake. Caries Res. 1997;31(2):148-54.

6. Kimoto M, Kishino M, Yura Y, Ogawa Y. A role of salivary carbonic anhydrase VI in dental plaque. Arch Oral Biol. 2006 Feb;51(2):117-22.

7. [Kulhavá L](https://www.proquest.com/indexinglinkhandler/sng/author/Kulhav$e1,+L/$N;jsessionid=7492B0E9966C0252B56BC30CD719B643.i-0cc096a45b7d4309a), [Eckhardt A](https://www.proquest.com/indexinglinkhandler/sng/author/Eckhardt,+A/$N;jsessionid=7492B0E9966C0252B56BC30CD719B643.i-0cc096a45b7d4309a), [Pataridis S](https://www.proquest.com/indexinglinkhandler/sng/author/Pataridis,+S/$N;jsessionid=7492B0E9966C0252B56BC30CD719B643.i-0cc096a45b7d4309a), [Bartoš M](https://www.proquest.com/indexinglinkhandler/sng/author/Barto$x0161,+M/$N;jsessionid=7492B0E9966C0252B56BC30CD719B643.i-0cc096a45b7d4309a), [Foltán R](https://www.proquest.com/indexinglinkhandler/sng/author/Folt$e1n,+R/$N;jsessionid=7492B0E9966C0252B56BC30CD719B643.i-0cc096a45b7d4309a), Mikšík I. Differences of Saliva Composition in Relation to Tooth Decay and Gender. [Folia Biologica](https://www.proquest.com/pubidlinkhandler/sng/pubtitle/Folia+Biologica/$N/32484/OpenView/2191313796/$B/3E7F59C731AF4D89PQ/1;jsessionid=7492B0E9966C0252B56BC30CD719B643.i-0cc096a45b7d4309a). 2018; 64(5/6): 195-203.

8. Orstavik D, Brandtzaeg P. Secretion of parotid IgA in relation to gingival inflammation and dental caries experience in man. Arch Oral Biol. 1975 Nov;20(11):701-4.

9. Patil V, Deshp RR, Chhabra RS, Kamath A, Patil D, Dungarwal P, Bagde KK, Shep S Kotwal, V. Comparative evaluation of salivary total protein concentration in male and female children in deciduous dentition. Research J Pharmaceutical, Biological Chemical Sci.2016; 7(2):1616-1619.

10. Pekovic DD, Adamkiewicz VW, Gornitsky M. Immunoglobulins in human dental caries. Arch Oral Biol. 1988;33(2):135-41.
