# Supplementary material for: Comparison of oral cavity protein abundance among caries-free and caries-affected individuals—a systematic review and meta-analysis
Source: Front Oral Health. 2023 Sep 15;4:1265817. doi: 10.3389/froh.2023.1265817 (PMC10540632; doi:10.3389/froh.2023.1265817)
Supplement: Supplementary file 2 [file Table2.docx]

**Table S2.** Total protein concentration in the oral cavity of caries-free and caries-affected individuals

| **Study (year)** | **Country** | **Participants (n)**  **[Age; mean ± sd]** | **Criteria for caries diagnosis** | **Caries experience** | **Clinical sample** | **Method** | **Results** | **Quality** |
| --- | --- | --- | --- | --- | --- | --- | --- | --- |
| Ahmadi-Motamayel et al. (2013b) | Iran | Caries-free (50)  [15 – 17 years-old]  Caries-active (50)  [15 – 17 years-old] | WHO | Caries-free: DMFS=0  Caries-active: DS ≥ 5 | Unstimulated saliva (expectoration; morning; 1.5 h fasting and 1.5h w.o tootbrushing) | Bradford | Total proteins (mg/dL; mean ± sd):  Caries-free= 787. 3± 677.7  Caries-active= 887.8 ± 610.4  (p=0.438) | FAIR |
| Aliakbarpour et al. (2021) | Iran | Caries-free (30)  [3-5 years-old]  ECC (30)  [3-5 years-old]  S-ECC (30)  [3-5 years-old] | Not informed | Not informed | Unstimulated saliva (morning; 1h fasting and 1h w.o toothbrushing) | Bradford | Total proteins (mg/dL; mean ± sd):  Caries-free= 295.39 ± 77.29*  ECC= 405.56 ± 94.75  S-ECC= 435.84 ± 109.74  ***(p<0.05)**  *Calculated (mean±sd) for*  *ECC and S-ECCs= 420.7±102.78* | FAIR |
| Araújo et al.  (2020) | Brazil | Caries-free (30)  [17.8 ± 4.5 months-old]  Early carious lesions (30)  [23.6 ± 5:6 months-old]  Moderate carious lesions (30)  [32.6 ± 4.5 months-old]  Advanced carious lesions (30)  [35.0 ± 2.8 months-old] | ICCMS | Number of lesions:  Caries-free= 0  Early carious lesions= 1.93±0.94  Mod. carious lesions= 1.83±0.87  Adv. carious lesion= 2.57±1.10 | Unstimulated saliva (cotton swab; morning; 2h fasting) | Lowry | Total protein (mg/dL) by mg protein concentration:  Adv. Carious lesion group > mod. Carious lesion >early carious lesion = caries-free  **(p<0.001)**  A positive but moderate correlation between the amount of salivary proteins and caries severity (Spearman’s r = 0.7084**, p < 0.0001**) | GOOD |
| Balekjian et al. (1975) | USA | Caries-resistant (11)  [from 17 to 21 years-old]  Caries susceptible (10)  [from 17 to 21 years-old] | WHO | Caries-susceptible: DMFT from 11 to 30; mean of 16.5 | Stimulated parotid saliva (cannulation) | Lowry | Total proteins (mg/dL; mean ± sd):  Caries-resistant= 110.1±38.1  Caries-susceptible= 131.6±26.8  (p>0.05) | FAIR |
| de Farias; Bezerra (2003) | Brazil | Caries-free (20)  [39.5±7.12 months]  ECC (20)  [37.7±9.05 months) | WHO | Caries-free: dmfs=0  ECC: dfms= 16.4±8.9. | Unstimulated saliva (aspiration; morning; 1h fasting and 1h after toothbrushing) | Red-molybdate method | Total proteins (mg/dL; mean ± sd):  Caries-free= 60.80±23.20  ECC= 63.70±34.60  (p>0.05) | FAIR |
| Doods et al.  (1997) | USA | Caries-free (38)  [mean 23.3 years-old]  Caries-active (49)  [mean 24.4 years-old] | NIDRC | Caries active: DMFS > 5 | Stimulated saliva – parotid (chemical) | Arneberg method | Total protein (mg/mL; mean ± sem):  Caries-free= 2.49±0.20  Caries-active=2.90±0.19  (p>0.05) | GOOD |

**Table S2 (cont).** Total protein concentration in the oral cavity of caries-free and caries-affected individuals

| **Study (year)** | **Country** | **Participants (n)**  **[Age; mean ± sd]** | **Criteria for caries diagnosis** | **Caries experience** | **Clinical sample** | **Method** | **Results** | **Quality** |
| --- | --- | --- | --- | --- | --- | --- | --- | --- |
| Hedenbjörk-Lager et al.  (2015) | Sweden | Caries-free (306)  [mean 50.6 ± 17.0 years-old]  Moderate caries (104)  [mean 50.6 ± 17.0 years-old]  High caries (41)  [mean 50.6 ± 17.0 years-old] | WHO | Caries-free: DS=0  Moderate caries: DS:1-2  High caries: DS≥3 | Stimulated saliva (chewing) | Bradford | Total proteins (mg/dL; mean ± sd):  Caries-free= 79.44±39.72 (n=299)  Moderate caries= 82.62±40.35 (n=102)  High-caries= 101.06±62.94 (n=40)  **(p=0.011)**  *Calculated (mean±sd) for*  *moderate-high caries = 91.84 ± 48.28* | FAIR |
| Lertsirivorakul et al. (2015) | Thailand | Caries-free (32)  [56.9 ± 7.2 months-old]  ECC (32)  [57.8 ± 8.2 months-old] | WHO | Caries-free: dmft=0  ECC: dmft= 12.5 ± 3.2  dmfs= 26.8 ± 15.5 | Unstimulated saliva (morning) | Lowry | Total proteins (mg/dL; mean ± sd):  Caries-free= 120 ± 30  ECC= 120 ± 40  (p=0.98) | FAIR |
| Letieri et al.  (2019) | Brazil | Caries-free (23)  [3.7 ± 1.2 years]  ECC (23)  [3.0 ± 1.0 years] | WHO (incl. non-cavitated) | ECC: dmfs= 10.2 (from 1 to 32) | Unstimulated saliva (aspiration; morning) | Bradford | Total proteins (mg/dL; mean ± sd):  Caries-free= 3.983±2.319  ECC= 3.814±2.489  (p>0.05) | FAIR |
| Mahjoub et al.  (2014) | Iran | Caries-free (40)  [3-5 years-old]  S-ECC (40)  [3-5 years-old] | WHO | Caries-free? dmfs=0  S-ECC: dmfs ≥ 4 | Unstimulated saliva (fasting) | Bradford | Total protein (mg/dL; mean ± sd):  Caries-free= 270.14±86.22  S-ECC= 323.18±128.71  (**p=0.033)** | GOOD |
| Mandel et al.  (1965) | US | Caries-immune (63)  [male median 29 years-old  female median 27 years-old]  Caries-active (63)  [male median 29 years-old  female median 27 years-old] | Not informed | Not informed | Stimulated saliva (sugar-citric acid lozenge; expectoration) | Folin-Ciocalteu method | Total proteins (mg/dL; mean ± sd):  *Calculated means for*  *Caries-free= 214±74.08*  *Caries-active= 206.5±71.18*  *(p>0.05)* | POOR |
| Murugeshappa et al. (2008) | India | Caries-free (75)  [7-12 years-old]  Caries active (35)  [7-12 years-old] | WHO | Caries active: DMF= 6.26 (DMFT≥5) | Unstimulated saliva (1h fasting) | Bradford | Total proteins (mg/dL; mean):  Caries-free=180.0  Caries-active=271.0  **(p<0.01)** | GOOD |
| Nireeksha et al.  (2017) | India | Caries-free (20)  [25-40 years-old]  Low caries (20)  [25-40 years-old]  Moderate caries (20)  [25-40 years-old]  High caries (20)  [25-40 years-old] | WHO | Caries-free: DMFT=0  Low caries: DMFT=1-3  Moderate caries: DMFT=4-10  High-caries: DMFT>10 | Unstimulated saliva (drooling; morning; 2h fasting) | Biuret reaction | Total proteins (mg/dL; mean ± sd):  Caries-free= 2040.0 ± 258.0  Low-caries= 1710.0 ± 417.0  Moderate caries= 820.0 ± 206.0  High-caries= 510.0 ± 164.0  **(p<0.001)**  *Calculated (mean±sd) for*  *Low-moderate-high caries = 887.5±584.29* | FAIR |

**Table S2 (cont).** Total protein concentration in the oral cavity of caries-free and caries-affected individuals

| **Study (year)** | **Country** | **Participants (n)**  **[Age; mean ± sd]** | **Criteria for caries diagnosis** | **Caries experience** | **Clinical sample** | **Method** | **Results** | **Quality** |
| --- | --- | --- | --- | --- | --- | --- | --- | --- |
| Pandey et al. (2015) | India | Caries-free (60)  [7-10 years-old (n=30)]  [11-15 years-old (n=30)]  Caries-active (60)  [7-10 years-old (n=30)]  [11-15 years-old (n=30)] | WHO | Caries-free: DMFS=0  Caries-actove: DMFS/dfs ≥ 5 | Unstimulated saliva (spiting, morning, 2h fasting) | Biuret reaction | Total proteins (mg/dL; mean ± sd):  Caries-free:  [7-10 years-old]  Girls: 350±80*  Boys: 320±110*  [11-15 years-old]  Girls: 430±10  Boys: 350±120*  Caries-active:  [7-10 years-old]  Girls: 430±140*  Boys: 440±130*  [11-15 years-old]  Girls: 380±90  Boys: 460±160*  * **(p<0.05)**  *Calculated means(male and female)*  *Caries-free (n=60)*  *370*±*10)*  *Caries-active (n=60)*  *425*±*120* | GOOD |
| Phattarataratip et al. (2011) | USA | Caries-free (30)  [13 years-old]  Caries-active (30)  [13 years-old] | WHO | Caries-active: 3 or more carious or filled surfaces (D2-3F). | Stimulated saliva (chewing) | BCA assay | Total protein (mg/dL; mean ± sd):  Caries-free=85.21±20.96  Caries-active=108,01±30.63  **(p=0.02)** | FAIR |

**Table S2 (cont).** Total protein concentration in the oral cavity of caries-free and caries-affected individuals

| **Study (year)** | **Country** | **Participants (n)**  **[Age; mean ± sd]** | **Criteria for caries diagnosis** | **Caries experience** | **Clinical sample** | **Method** | **Results** | **Quality** |
| --- | --- | --- | --- | --- | --- | --- | --- | --- |
| Preethi et al.  (2010) | India | Caries-free (60)  [7-10 years-old (n=30)  11-14 years-old (n=30)]  Caries-active (60)  [7-10 years-old (n=30)  11-14 years-old (n=30)] | WHO | Not informed | Unstimulated saliva (aspiration) | Not informed | Total protein (mg/dL; mean ± sd):  Caries-free:  [7-10 years-old]  Girls: 5.68 ± 1.33  Boys: 5.59 ± 1.34*  [11-14 years-old]  Girls: 5.57 ± 1.19**  Boys: 5.28 ± 0.84 ***  Caries-active:  [7-10 years-old]  Girls: 6.61 ± 1.30  Boys: 7.23 ± 1.37*  [11-14 years-old]  Girls: 6.57 ± 1.50**  Boys: 7.36 ± 1.52***  */**/*** **(p<0.05)**  *Calculated means (7-14 years):*  *Caries-free= 5.53±1.16 (n=60);*  *Caries-active= 6.94±1.42 (n=60)* | FAIR |
| Razi et al.  (2020) | India | Caries-free (20)  [12-15 years-old]  Caries-active (20)  [12-15 years-old] | WHO | Caries-free: DMFS=0  Caries-active: DMFS≥10 | Unstimulated saliva (drooling; morning; 1.5h fasting) | Lowry | Total proteins (mg/dL; mean ± sd):  Caries-free= 289.0±11.0  Caries-active= 328.0±12.0  **(p=0.015)** | GOOD |
| Roa et al.  (2008) | Colombia | Caries-free (49)  [> 18 years-old]  History of caries (49)  [> 18 years-old]  Caries active (47)  [> 18 years-old] | WHO | History of caries: with amalgam or resin fillings and currently free of  caries  Caries active: with multiple cavities, including enamel and dentine. | Unstimulated saliva (drooling; 2h fasting) | Bradford | Total protein (mg/dL; mean ± sd):  Caries-free= 119.6±36.24  History of caries=119.8±29.98  Caries-active= 126.2±31.1  (p=0.4204)  *Calculated means for caries: 123.0±30.54* | FAIR |

**Table S2 (cont).** Total protein concentration in the oral cavity of caries-free and caries-affected individuals

| **Study (year)** | **Country** | **Participants (n)**  **[Age; mean ± sd]** | **Criteria for caries diagnosis** | **Caries experience** | **Clinical sample** | **Method** | **Results** | **Quality** |
| --- | --- | --- | --- | --- | --- | --- | --- | --- |
| Silva et al.  (2016) | Brazil | Caries-free (30)  [0-3 years-old]  S-ECC (30)  [0-3 years-old] | WHO | Caries-free: dfms=0  S-ECC: dmfs= 3.7±3.44 | Unstimulated saliva (expectoration; morning; 2h fasting] | Lowry | Total protein (mg/dL; mean ± sd):  Caries-free= 7.0±1.2  S-ECC= 8.3±2.0  **(p<0.01)** | GOOD |
| Tulunoglu et al.  (2006) | Turkey | Caries-free (40)  [7-10 years-old (n=20)  11-15 years-old (n=20)]  Caries-active (40)  [7-10 years-old (n=20)  11-15 years-old (n=20)] | WHO | Caries-free: DMFT=0  Caries-active: DMFT≥5 | Unstimulated saliva (morning; 2h fasting) | Biuret reaction | Total protein (mg/dL; mean ± sd):  Caries-free:  [7-10 years-old]  Girls 330.0±80.0  Boys: 350.0±140.0  [11-15 years-old]  Girls 520.0±100.0  Boys: 450.0±140.0  Caries-active:  [7-10 years-old]  Girls: 350.0±90.0  Boys: 650.0±500.0  [11-15 years-old]  Girls: 430.0±430.0  Boys: 480.0±160.0  (p>0.05)  *Calculated means (7-15 years):*  *Caries-free=* *412.5±137.47 (n=40);*  *Caries-active=* *477.5±350.61 (n=40)* | FAIR |
| Zengo et al.  (1971) | USA | Caries resistant (20)  [adults]  Caries susceptible (20)  [adults] | Not informed | Caries-free: DF=0  Caries susceptible: DMF≥15 | Stimulated saliva  (citric acid; morning; 2h fasting) | Biuret reaction | Total protein (mg/dL; mean ± sd):  Stimulated parotid saliva:  Caries-resistant: 316 ± 200  Caries-susceptible: 260 ±128  **(p<0.05)**  Stimulated submaxillary saliva:  Caries-resistant: 165 ± 65  Caries-susceptible: 197 ± 70  (p>0.05) | POOR |
