# Supplementary material for: Comparison of oral cavity protein abundance among caries-free and caries-affected individuals—a systematic review and meta-analysis
Source: Front Oral Health. 2023 Sep 15;4:1265817. doi: 10.3389/froh.2023.1265817 (PMC10540632; doi:10.3389/froh.2023.1265817)
Supplement: Supplementary file 3 [file Table3.docx]

**Table S3.** Total salivary antioxidant capacity in the oral cavity of caries-free and caries-affected individuals

| **Study (year)** | **Country** | **Participants (n)**  **[Age; mean ± sd]** | **Criteria for caries diagnosis** | **Caries experience** | **Clinical sample** | **Method** | **Results** | **Quality** |
| --- | --- | --- | --- | --- | --- | --- | --- | --- |
| Ahmadi-Motamayel et al.  (2013a) | Iran | Caries-free (50)  [15-17 years-old]  Caries active (50)  [15-17 years-old] | WHO | Caries-free: DMFT=0  Caries active: DS≥5 | Unstimulated saliva (expectoration; morning; 1.5h fasting) | ABTS method | TAC (mmol/L; mean ± sd):  Caries-free= 40.74±10.85  Caries-active= 50.53±16.69  **(p<0.001)** | FAIR |
| Ahmadi-Motamayel et al.  (2018) | Iran | Caries-free (56)  [15-19 years-old]  Caries active (62)  [15-19 years-old] | WHO | Caries-free: DMFT=0  Caries active: DMFT≥5 | Unstimulated saliva (expectoration; morning) | FRAP assay | TAC (mmol/L; mean ± sd):  Caries-free= 0.17 ± 0.01  Caries-active= 0.17 ± 0.02  (p=0.07) | GOOD |
| Aliakbarpour et al. (2021) | Iran | Caries-free (30)  [3-5 years-old]  ECC (30)  [3-5 years-old]  S-ECC (30)  [3-5 years-old] | Not informed | Not informed | Unstimulated saliva (morning; 1h fasting and wo. toothbrushing) | TBARS assay | TAC (mmol/L; mean ± sd):  Caries-free= 0,000054 ± 0,000029*  ECC= 0,000098 ± 0,000034  S-ECC= 0,000109 ± 0,000036  ***(p<0.05)**  *Calculated (mean±sd) for*  *ECC and S-ECCs= 0.0001035±0.0000351* | FAIR |
| Araújo et al.  (2020) | Brazil | Caries-free (30)  [17.8 ± 4.5 months-old]  Early carious lesions (30)  [23.6 ± 5:6 months-old]  Moderate carious lesions (30)  [32.6 ± 4.5 months-old]  Advanced carious lesions (30)  [35.0 ± 2.8 months-old] | ICCMS | Number of lesions:  Caries-free= 0  Early carious lesions= 1.93±0.94  Mod. carious lesions= 1.83±0.87  Adv. carious lesion= 2.57 ± 1.10 | Unstimulated saliva (cotton swab; morning; 2h fasting) | FRAP assay | TAC (umol/L) by mg protein concentration:    Adv. Carious lesion group > mod. Carious lesion >early carious lesion = caries-free  **(p<0.001)**  A positive and strong correlation was observed between caries severity and TAC (Spearman’s r = 0.8425**, p < 0.0001**) | GOOD |
| Banda et al.  (2017) | India | Caries-free (30)  [7.13 ± 0.86 years-old]  Caries (30)  [8.03 ± 1.03 years-old] | WHO | Caries-free: D/d=0  Caries: D/d > 4 | Unstimulated saliva (spiting; morning; 1h fasting) | Molybdenium method | TAL (mmol/L; mean ± sd):  Caries-free= 0.0063±0.02305  Caries= 0.1102±0.07101  **(p<0.0001)** | GOOD |
| Hedge et al.  (2009) | India | Caries-free (25)  [4.44±0.58 years-old]  ECC (25)  [4.36±0.70 years-old]  Caries-free (25)  [7.80±0.82 years-old]  Rampant caries (25)  [7.56±1.04 years-old] | WHO | Not informed | Unstimulated saliva (spiting) | TBARS assay | TAC (mmol/L; mean ± sd):  Caries-free= 0.014±0.004  ECC=0.025±0.012  **(p<0.001)**  Caries-free= 0.022±0.004  Rampant caries= 0.046±0.0009  **(p<0.001)** | FAIR |

**Table S3 (cont).** Total salivary antioxidant capacity in the oral cavity of caries-free and caries-affected individuals

| **Study (year)** | **Country** | **Participants (n)**  **[Age; mean ± sd]** | **Criteria for caries diagnosis** | **Caries experience** | **Clinical sample** | **Method** | **Results** | **Quality** |
| --- | --- | --- | --- | --- | --- | --- | --- | --- |
| Hedge et al.  (2013a) | India | Caries-free (25)  [25-50 years-old]  Low caries (25)  [25-50 years-old]  Moderate caries (25)  [25-50 years-old]  High caries (25)  [25-50 years-old] | WHO | Caries-free: DMFT=0  Low caries: (0<DMFT<3  Moderate caries: 3<DMFT<10  High caries: DMFT>10 | Not clear if unstimulated (drooling) of stimulated (chewing) saliva | Molybdenium method | TAC (mmol/L; mean ± sd):  Caries-free= 0.00034 ± 0.00013  Low-caries= 0.00044 ± 0.00013  Moderate caries= 0.00056 ± 0.00026  High caries= 0.00059 ± 0.00018  **(p<0.001)**  *Calculated (mean±sd) for*  *Low-moderate-high caries=*  *0.000545±0.0002* | FAIR |
| Jurczak et al.  (2017) | Poland | Caries-free (27)  [3.11 ± 1.19 years-old]  Non-cavitated group (26)  [2.35 ±1.09 years-old]  Extensive decay (26)  [3.56 ±1.25 years-old] | ICDAS II | Caries-free: ICDAS II=0  Non-cavitated group:  1–2 ICDAS II; 2.35 ± 1.09  Extensive decay:  5–6 ICDAS II; 3.56 ± 1.25 | Unstimulated saliva (cotton swab; morning; fasting) | FRAP assay | TAC (mmol/L; median; lower-upper quartile):  Extensive decay: 1.73 (1.72–1.74)  Extensive decay > non-cavitated group > caries-free **(p<0.001)** | GOOD |
| Mahjoub et al.  (2014) | Iran | Caries-free (40)  [3-5 years-old]  S-ECC (40)  [3-5 years-old] | WHO | Caries-free? dmfs=0  S-ECC: dmfs ≥ 4 | Unstimulated saliva (fasting) | FRAP assay | TAC (mmol/L; mean ± sd):  Caries-free= 0.579±0.145  S-ECC= 0.647±0.121  (**p=0.025)**  A positive correlation between salivary TAC and dmfs scores in S-ECC children by Pearson’s correlation test (r = 0.725, **p <** **0.001)** | GOOD |
| Muchandi et al.  (2015) | India | Caries-free (25)  [3-5 years-old]  S-ECC (25)  [3-5 years-old] | Not informed | Not informed | Unstimulated saliva (drooling) | TBARS assay | TAC (mmol/L; mean ± sd):  Caries-free= 1.08 ± 0.17  S-ECC= 1.82 ± 0.19  (**p<0.0001)** | FAIR |

**Table S3 (cont).** Total salivary antioxidant capacity in the oral cavity of caries-free and caries-affected individuals

| **Study (year)** | **Country** | **Participants (n)**  **[Age; mean ± sd]** | **Criteria for caries diagnosis** | **Caries experience** | **Clinical sample** | **Method** | **Results** | **Quality** |
| --- | --- | --- | --- | --- | --- | --- | --- | --- |
| Pandey et al.  (2015) | India | Caries-free (60)  [7-10 years-old (n=30)]  [11-15 years-old (n=30)]  Caries-active (60)  [7-10 years-old (n=30)]  [11-15 years-old (n=30)] | WHO | Caries-free: DMFS=0  Caries-active: DMFS/dfs ≥ 5 | Unstimulated saliva (spiting, morning, 2h fasting) | FRAP assay | TAC (mmol/L; mean ± sd):  Caries-free:  [7-10 years-old]  Girls: 0.42±0.08*  Boys: 0.36±0.37*  [11-15 years-old]  Girls: 0.46±0.09  Boys: 0.38±0.06*  Caries-active:  [7-10 years-old]  Girls: 0.46±0.10*  Boys: 0.47±0.11*  [11-15 years-old]  Girls: 0.40±0.19  Boys: 0.48±0.12*  * **(p<0.05)**  *Calculated means(male and female)*  *Caries-free (n=60)*  *0.40*±0.*10*  *Caries-active (n=60)*  *0.45*±*0.12* | GOOD |
| Patel; Pujara  (2015) | India | Group A  Caries-free (25)  [below 71 months-old]  ECC (25)  [below 71 months-old (25)]  Group B  Caries-free (25)  [from 6-10 years-old]  ECC (25)  [from 6-10 years-old] | WHO | Group A  Caries-free: dft=0;  ECC: dft=3.32±1.74;  Group B  Caries-free: DMFT=0  ECC: DMFT= 6.16±0.98 | Unstimulated saliva (expectoration) | Spectrophotometric method | Group A  TAC (mmol/L; mean ± sd):  Caries-free= 0.014152±0.004246  ECC= 0.025584±0.012123  **p<0.0001**  Group B  TAC (mmol/L; mean ± sd):  Caries-free= 0.02296±0.00476  ECC=0.04612±0.00099  **p<0.0001** | FAIR |

**Table S3 (cont).** Total salivary antioxidant capacity in the oral cavity of caries-free and caries-affected individuals

| **Study (year)** | **Country** | **Participants (n)**  **[Age; mean ± sd]** | **Criteria for caries diagnosis** | **Caries experience** | **Clinical sample** | **Method** | **Results** | **Quality** |
| --- | --- | --- | --- | --- | --- | --- | --- | --- |
| Preethi et al.  (2010) | India | Caries-free (60)  [7-10 years-old (n=30)  11-14 years-old (n=30)]  Caries-active (60)  [7-10 years-old (n=30)  11-14 years-old (n=30)] | WHO | Not informed | Unstimulated saliva (aspiration) | TBARS assay | TAC (mmol/L; mean ± sd):  Caries-free:  [7-10 years-old]  Girls: 0.00016 ± 0.00003*  Boys: 0.00016 ± 0.00005**  [11-14 years-old]  Girls: 0.00019 ± 0.00004***  Boys: 0.00019 ± 0.00004****  Caries-active:  [7-10 years-old]  Girls: 0.00023 ± 0.00005*  Boys: 0.00020 ± 0.00004**  [11-14 years-old]  Girls: 0.00023 ± 0.00004***  Boys: 0.00022 ± 0.00004****  */**/***/**** **(p<0.05)**  *Calculated means (7-14 years):*  *Caries-free=0.000175±0.00004199 (n=60); Caries-active=0.00022±0.00004338 (n=60)* | FAIR |
| Pyati et al.  (2018) | India | Caries-free (50)  [6-12 years-old]  Caries-active (50)  [6-12 years-old] | WHO | Caries-free: DMFS/dfs=0  Caries active:  DMFS/dfs=6.26 ± 0.90 | Unstimulated saliva (aspiration; morning; 1h fasting) | TBARS assay | TAC (mmol/L; mean ± sd):  Caries-free= 0.35 ± 0.16  Caries active= 0.44 ± 0.17  **(p <0.006)** | GOOD |
| Rahmani et al.  (2016) | Iran | Caries-free (60)  [14-18 years-old]  Caries (60)  [14-18 years-old] | WHO | Caries-free: DMFT=0  Caries: DMFT ≥5 | Unstimulated saliva (drooling; morning; 1.5h fasting) | FRAP assay | TAC (mmol/L; mean ± sd):  Caries-free= 0.000396 ± 0.000077  Caries= 0.000256 ± 0.000106  **(p<0.001)** | GOOD |

**Table S3 (cont).** Total salivary antioxidant capacity in the oral cavity of caries-free and caries-affected individuals

| **Study (year)** | **Country** | **Participants (n)**  **[Age; mean ± sd]** | **Criteria for caries diagnosis** | **Caries experience** | **Clinical sample** | **Method** | **Results** | **Quality** |
| --- | --- | --- | --- | --- | --- | --- | --- | --- |
| Salman et al.  (2021) | Iran | Caries-free (84)  [3-18 years-old]  Caries-active (78)  [3-18 years-old] | WHO | Caries-free: Caries index=0  Caries active: Caries index=2 | Unstimulated saliva (morning) | TBARS assay | TAC (mmol/L; mean ± sd):  Caries-free:  [3-12 years-old]  0,494 ± 0,229  [13-18 years-old]  0,675 ± 0,163*  [3-18 years-old]  0,541 ± 0,228  Caries-active  [3-12 years-old]  0,520 ± 0,216  [13-18 years-old]  0,530 ± 0,166*  [3-18 years-old]  0,522 ± 0,204  **(*p<0,05)** | FAIR |
| Shaki et al.  (2020) | Iran | Caries-free (40)  [3-5 years-old]  Caries-active (40)  [3-5 years-old] | WHO | Caries-free: DMFT=0  Caries-active: DMFT ≥5 | Unstimulated saliva (expectoration; morning; 2h fasting) | FRAP asaay | Salivary total antioxidant capacity (FRAP/FeSO4µM) higher in caries active rather than in caries-free  **(p<0.05)** | FAIR |
| Silva et al.  (2016) | Brazil | Caries-free (30)  [0-3 years-old]  S-ECC (30)  [0-3 years-old] | WHO | Caries-free: dfms=0  S-ECC: dmfs= 3.7±3.44 | Unstimulated saliva (expectoration; morning; 2h fasting] | FRAP assay | TAC (mmol/L; mean ± sd):  Caries-free= 49.1 ± 23.24  S-ECC= 61.5 ± 20.18  **(p<0.05)** | GOOD |

**Table S3 (cont).** Total salivary antioxidant capacity in the oral cavity of caries-free and caries-affected individuals

| **Study (year)** | **Country** | **Participants (n)**  **[Age; mean ± sd]** | **Criteria for caries diagnosis** | **Caries experience** | **Clinical sample** | **Method** | **Results** | **Quality** |
| --- | --- | --- | --- | --- | --- | --- | --- | --- |
| Tulunoglu et al.  (2006) | Turkey | Caries-free (40)  [7-10 years-old (n=20)  11-15 years-old (n=20)]  Caries-active (40)  [7-10 years-old (n=20)  11-15 years-old (n=20)] | WHO | Caries-free: DMFT=0  Caries-active: DMFT≥5 | Unstimulated saliva (morning; 2h fasting) | ABTS method | TAC (mmol/L; mean ± sd):  Caries-free:  [7-10 years-old]  Girls 0.39 ± 0.08  Boys: 0.48 ± 0.20  [11-15 years-old]  Girls 0.65 ± 0.16  Boys: 0.56 ± 0.30  Caries-active:  [7-10 years-old]  Girls: 0.45 ± 0.13  Boys: 0.60 ± 0.15  [11-15 years-old]  Girls: 0.57 ± 0.18  Boys: 0.69 ± 0.25  (p>0.05)  *Calculated means (7-15 years):*  *Caries-free=* *0.52±0.21 (n=40);*  *Caries-active=* *0.57±0.19 (n=40)* | FAIR |

FRAP: Ferric Reducing Antioxidant Power; TBARS: Thiobarbituric Acid Reactive Substances; ABTS: 2,2-azino-di (3-ethylbenzthiazoline-6-sulphonate)
