# Supplementary material for: Comparison of oral cavity protein abundance among caries-free and caries-affected individuals—a systematic review and meta-analysis
Source: Front Oral Health. 2023 Sep 15;4:1265817. doi: 10.3389/froh.2023.1265817 (PMC10540632; doi:10.3389/froh.2023.1265817)
Supplement: Supplementary file 4 [file Table4.docx]

**Table S4.** Concentration of immunoglobulins (s-IgA and IgG) in the oral cavity of caries-free and caries-affected individuals

| **Study (year)** | **Country** | **Participants (n)**  **[Age; mean ± sd]** | **Criteria for caries diagnosis** | **Caries experience** | **Clinical sample** | **Method** | **Results** | **Quality** |
| --- | --- | --- | --- | --- | --- | --- | --- | --- |
| Ahmad et al. (2021) | India | Caries-free (50)  [8-12 years old]  Caries-active (50)  [8-12 years-old] | WHO | Caries-free: DMFT or dmft=0  Caries-active: DMFT or dmft ≥ 5 | Unstimulated saliva  (drooling; morning; 1h fasting) | ELISA | s-IgA (mg/dL; mean ± sd):  Caries-free= 0.562 ± 0.177  Caries active= 0.298 ± 0.166  **(p=0.001)** | GOOD |
| Al- Amoudi et al. (2007) | Saudi Arabia | Caries-free (30)  S-ECC (30)  [3-5 years; 4.23 ± 0.65 years) | WHO | Caries-free: dmft=0  S-ECC: dmft =8.83 ± 3.37 | Stimulated saliva  (morning; 1h fasting and without previous toothbrusing) | ELISA | s-IgA (ug; mean ± sd):  Caries-free= 29.8±33.9  S-ECC=82.0±67.6  **(p<0.001)** | GOOD |
| Alaluusua et al. (1983)* | Finland | Caries-free (23)  Caries-susceptible (16)  [11-13 months at baseline; up to 4 years at the follow-up] | Not informed | Caries-free: dmfs=0  Caries-susceptible:  dmfs= 0.1 ± 0.5 (at 2 years); dmfs= 2.4 ± 3.5 (at 3 years); dmfs = 5.9 ± 4.2 (at 4 years) | Unstimulated saliva (morning; 1-3h after breakfast) | Radial Immuno-diffusion technique | s-IgA (g/L; geometric mean):  Caries-free: 0.044 (2-years follow-up)  Caries-susceptible: 0.073 (2-years follow-up)  **(p<0.05**)  No differences were found for the other  time points | GOOD |
| Bagherian et al. (2008) | Iran | Caries-free (45)  [59.4 ± 12.09 months]  S-ECC (45)  [60.9± 8.8 months] | WHO | Caries-free: dmft=0  [59.4±12.09 months]  S-ECC: dmft= 9.3±3.6  [60.9± 8.8 months] | Unstimulated saliva (expectoration; morning; 1.5h fasting; 1.5h after toothbrushing) | ELISA | s-IgA (mg/dL; mean ± sd):  Caries-free= 148.45±81.16;  S-ECC= 196.14±100.07  **(p=0.015)** | GOOD |
| Castro et al.  (2016) | Chile | Caries-free (20)  [24 ± 2 years old]  Dental caries (20)  [25 ± 3 years old] | ICDAS | Caries-free: DMFT=0  Dental caries: DMFT=3.9 ± 0.7 (at least 3 lesions ICDAS codes 5 or 6) | Unstimulated saliva (drooling; morning; 2h fasting) | Western blotting | s-IgA (ug; mean ± sd):  Caries-free= 11.27±0.5;  Dental caries = 1.71±0.2  **(p=0.001)** | GOOD |
| Chawda et al. (2010) | India | Caries-free(10)  Low caries (10)  High caries (10)  [4-8 years-old] | WHO | Caries free: DMFT/dmft=0  Low caries: DMFT and/or dft= 1-5;  High caries: DMFT and/or dft= 6-10 | Unstimulated saliva (expectoration; morning) | Immunoturbido-metry | s-IgA (mg/dL; mean ± sd):  Caries-free=24.36±4.87b  Low-caries=18.66±4.84a  High-caries: 16.63±3.04a  **(p<0.05)**  *Calculated (mean±sd) for*  *low-high caries= 17.64±4.06* | GOOD |

**Table S4 (cont).** Concentration of immunoglobulins (s-IgA and IgG) in the oral cavity of caries-free and caries-affected individuals

| **Study (year)** | **Country** | **Participants (n)**  **[Age; mean ± sd]** | **Criteria for caries diagnosis** | **Caries experience** | **Clinical sample** | **Method** | **Results** | **Quality** |
| --- | --- | --- | --- | --- | --- | --- | --- | --- |
| Colombo et al. (2016a) | Brazil | Caries-free (19)  [46.26±5.05 months]  ECC (17)  [45.94± 9.83 months]  S-ECC (21)  [48.10±8.59 months] | WHO  (incl. non-cavitated lesions) | Caries-free: dmfs=0  ECC: dmfs= 2.0±1.06 (cavt)  dmfs= 2.94±1.67 (n.cavt)  S-ECC: dmfs= 23.43±17.17 (cavt)  dfms= 28.28 ±18.76 (n. cavt) | Unstimulated saliva (drooling; afternoon; 1h fasting) | ELISA | s-IgA (ng/mL; median; range)  Caries-free= 99.15 (24.52–114.24)  ECC= 83.24 (32.27–114.56)  S-ECC 88.04 (18.17–109.31)  p= 0.125 | FAIR |
| de Farias; Bezerra (2003) | Brazil | Caries-free (20)  [39.5±7.12 months]  ECC (20)  [37.7±9.05 months) | WHO | Caries-free: dmfs=0  ECC: dfms= 16.4±8.9. | Unstimulated saliva (aspiration; morning; 1h fasting and 1h after toothbrusing) | Nephelometric technique | s-IgA (mg/dL; mean ± sd):  Caries-free= 3.25±2.10  ECC= 5.04±4.50  **(p<0.05)** | FAIR |
| Doifode; Damle  (2011) | India | Caries-free (15)  [9.13±0.35 years-old]  Caries active (15)  [8.73±0.46 years-old] | WHO | Caries-free: dfs=0  Caries active: dfs=19.00±8.52; DMFS=1.60 ± 2.03 | Unstimulated saliva | radial immune-diffusion method | s-IgA (mg/dL; mean ± sd):  Caries-free= 10.74±1.52  Caries active= 8.98±1.56  **(p=0.012)** | FAIR |
| Hedge et al.  (2013c) | India | Caries-free (20)  [20-30 years-old]  Low caries (20)  [20-30 years-old]  Moderate caries (20)  [20-30 years-old]  High caries (20)  [20-30 years-old] | WHO | Caries-free: DMFT=0  Low caries: DMFT 1-5  Moderate caries: DMFT 6-10  High caries: DMFT>10 | Unstimulated saliva (drooling; morning; 1h fasting) | immunoglobulin estimation kit | s-IgA (mg/dL; mean ± sd):  Caries-free= 315.51 ± 46.28  Low-caries= 294.61± 46.28  Moderate caries= 156.27± 46.28  High-caries= 120.65± 46.28d  **(p<0.001)**  *Calculated (mean±sd) for*  *Low-moderate-high caries = 190.51±88.28* | FAIR |
| Letieri et al.  (2019) | Brazil | Caries-free (23)  [3.7 ± 1.2 years]  ECC (23)  [3.0 ± 1.0 years] | WHO (incl. non-cavitated lesions) | ECC: dmfs= 10.2 (from 1 to 32) | Unstimulated saliva (aspiration; morning) | Indirect competitive immunoassay | s-IgA (mg/dL; mean ± sd):  Caries-free= 2.540±1.544  ECC=4.689±4.194  **(p=0.03)** | FAIR |
| Murugeshappa et al. (2008) | India | Caries-free (75)  [7-12 years-old]  Caries active (35)  [7-12 years-old] | WHO | Caries active: DMF= 6.26 (DMFT≥5) | Unstimulated saliva (1h fasting) | ELISA | s-IgA (mg/dL; mean):  Caries-free=0.01142  Caries-active=0.00708  **(p<0.01)** | GOOD |
| Naspitz et al.  (1999) | Brazil | Caries-free (20)  [3-5 years-old]  Caries lesions (15)  [3-5 years-old]  Rampant caries (14)  [3-5 years-old] | WHO | Caries-free: dmfs=0  Caries lesion: dmfs=1-2  Rampant caries: dmfs=12-46 | Unstimulated saliva (3h fasting) | ELISA | s-IgA (mg/dL; mean ± sd):  Caries-free=13.895±4.224  Caries lesions=14.6±6.870  Rampant caries=19.007±10.668  (p>0.05)  *Calculated (mean±sd) for*  *Caries lesion-rampant caries= 16.72±9.021* | POOR |

**Table S4 (cont).** Concentration of immunoglobulins (s-IgA and IgG) in the oral cavity of caries-free and caries-affected individuals

| **Study (year)** | **Country** | **Participants (n)**  **[Age; mean ± sd]** | **Criteria for caries diagnosis** | **Caries experience** | **Clinical sample** | **Method** | **Results** | **Quality** |
| --- | --- | --- | --- | --- | --- | --- | --- | --- |
| Nireeksha et al.  (2017) | India | Caries-free (20)  [25-40 years-old]  Low caries (20)  [25-40 years-old]  Moderate caries (20)  [25-40 years-old]  High caries (20)  [25-40 years-old] | WHO | Caries-free: DMFT=0  Low caries: DMFT=1-3  Moderate caries: DMFT=4-10  High-caries: DMFT>10 | Unstimulated saliva (drooling; morning; 2h fasting) | immunoglobulin estimation kit | s-IgA (mg/dL; mean ± sd):  Caries-free= 10.88± 0.628  Low-caries= 9.27±0.624  Moderate caries= 8.24±1.047  High-caries= 5.45± 0.767  **(p<0.001)**  *Calculated (mean±sd) for*  *Low-moderate-high caries = 7.65±1.81* | FAIR |
| Pandey et al.  (2018) | India | Caries-free (20)  [10.2 ± 2.35 years-old]  Low-caries (20)  [9.5 ± 2.51 years-old]  High-caries (20)  [9.214 ± 2.28 years-old] | WHO (incl. non-cavitated lesions) | Caries-free: CI=0  Low-caries: CI=2.32 ± 0.86  High-caries: CI= 6.74 ± 2.16 | Unstimulated saliva (expectoration; morning; fasting] | ELISA | s-IgA (mg/dL; mean ± sd):  Caries-free= 21.48±2.756  Low-caries= 18.610±2.470  High-caries= 14.253±2.24  **(p<0.001)**  *Calculated (mean±sd) for*  *Low caries-high caries= 16.43±3.20* | FAIR |
| Parisotto et al. (2011)** | Brazil | Caries-free (23)  [5 years-old]  Caries-active (17)  [5 years-old] | WHO (incl. non-cavitated lesions) | Caries-free: dfms=0  Caries-active: dmfs≥3 | Unstimulated saliva (drooling; afternoon; 1h fasting] | ELISA | s-IgA (mg/dL; mean ± sd):  Caries-free= 13.222 ±9.126  Caries-active= 18.197±14.092  (caries-free and caries-active not compared) | FAIR |
| Priya et al.  (2013)*** | India | Caries-free (15)  [7-12 years-old]  Caries active (15)  [7-12 years-old] | WHO | Caries-free: DMFT=0  Caries active: DMFT≥5 | Unstimulated saliva (drooling; 1h fasting) | ELISA | s-IgA (mg/dL; mean ± sd):  Caries-free= 1.19 ± 0.158  Caries-active= 1.307 ± 0.155  (P=0.05) | FAIR |
| Ranadheer et al.  (2011) | India | Caries-free (20)  [8-12 years-old]  Caries active (20)  [8-12 years-old] | WHO | Caries-free: DMFT=0  Caries active: DMFT≥3 (mean=4.2) | Unstimulated saliva (drooling;1h fasting) | ELISA | s-IgA (mg/dL; mean ± sd):  Caries-free= 7.585±2.48  Caries-active= 11.760±1.8  **(p<0.05)** | FAIR |
| Razi et al.  (2020) | India | Caries-free (20)  [12-15 years-old]  Caries-active (20)  [12-15 years-old] | WHO | Caries-free: DMFS=0  Caries-active: DMFS≥10 | Unstimulated saliva (drooling; morning; 1.5h fasting) | single radial immunodiffusion method | s-IgA (mg/dL; mean ± sd):  Caries-free= 10.63±2.85  Caries-active= 8.50±1.43  **(p=0.015)** | GOOD |
| Shifa et al.  (2008) | India | Caries-free (10)  [3-6 years-old]  Caries-active (10)  [3-6 years-old] | WHO | Caries-free: DMFT=0  Caries-active: DMFT≥5 | Unstimulated saliva (drooling; morning; 2h fasting) | Immunoturbidimetry | s-IgA (mg/dL; mean ± sd):  Caries-free= 229.9±43.19  Caries-active= 221.1±77.86  (p>0.05) | FAIR |

**Table S4 (cont).** Concentration of immunoglobulins (s-IgA and IgG) in the oral cavity of caries-free and caries-affected individuals

| **Study (year)** | **Country** | **Participants (n)**  **[Age; mean ± sd]** | **Criteria for caries diagnosis** | **Caries experience** | **Clinical sample** | **Method** | **Results** | **Quality** |
| --- | --- | --- | --- | --- | --- | --- | --- | --- |
| Stuchell; Mandel  (1978) | USA | Caries resistant (20)  [≥20 years-old]  Caries active (14)  [≥20 years-old] | WHO | Caries-resistant: DMFS=0  Caries-active: DMF 15-45 | Stimulated saliva (citric acid) | radial immune-diffusion method | s-IgA (mg/dL; mean ± sd):  Stimulated parotid saliva:  Caries-resistant: 5.3±1.6 (n=20)  Caries-active: 3.6±1.0 (n=14)  **(p<0.05)**  Stimulated submaxillary saliva:  Caries-resistant: 5.1±0.9 (n=20)  Caries-active: 4.0±0.06 (n=6)  **(p<0.05)** | POOR |
| Thaweboon et al.  (2008) | Thailand | Caries-free (15)  [92.73±19.86 months]  Rampant caries (15)  [92.46±5.05 months] | WHO | Caries-free: dmft=0  Rampant caries: dmft≥5; dmfs≥10 | Stimulated saliva (chewing; 2h fasting) | Enzyme immunoassay kit | s-IgA (mg/dL; mean ± sd):  Caries-free= 8.6473±4.323  Rampant-caries= 11.4964±3.424  **(p<0.05)** | FAIR |
| Yang et al.  (2015a) | China | Caries-free (36)  [3.57±0.40 years-old]  ECC (21)  [3.53±0.38 years-old]  S-ECC (13)  [3.51±0.41 years-old] | WHO | Caries-free: dfmt=0; dmfs=0  ECC: dmft=1.91±0.77;  dmfs=3.23 ± 2.37  S-ECC: dmft=(7.77 ± 4.11; dmfs=16.31 ± 12.54 | Unstimulated saliva (aspiration; morning; 1h fasting) | ELISA | s-IgA (mg/100 mg total protein; mean ± sd):  Caries-free= 23.4±30.39  ECC=20.7±24.69  S-ECC=60.1±79.37  **(p=0.014)** | GOOD |
| Zengo et al.  (1971) | USA | Caries resistant (20)  [adults]  Caries susceptible (20)  [adults] | Not informed | Caries-free: DF=0  Caries susceptible: DMF≥15 | Stimulated saliva  (citric acid; morning; 2h fasting) | radial immune-diffusion method | s-IgA (mg/dL; mean ± sd):  Stimulated parotid saliva:  Caries-resistant: 2.44 ±1.63  Caries-susceptible: 1.82 ± 0.88  (p>0.05)  Stimulated submaxillary saliva:  Caries-resistant: 165 ±65  Caries-susceptible: 197 ±70  **(p<0.05)** | POOR |

*Cohort (4-year follow-up data); **Cohort(at 1-year follow-up data); ***Cohort (baseline data)

**Table S4 (cont).** Concentration of immunoglobulins (s-IgA and IgG) in the oral cavity of caries-free and caries-affected individuals

| **Study (year)** | **Country** | **Participants (n)**  **[Age; mean ± sd]** | **Criteria for caries diagnosis** | **Caries experience** | **Clinical sample** | **Method** | **Results** | **Quality** |
| --- | --- | --- | --- | --- | --- | --- | --- | --- |
| Bagherian et al. (2008) | Iran | Caries-free (45)  [59.4 ± 12.09 months]  S-ECC (45)  [60.9± 8.8 months] | WHO (cavitation) | Caries-free: dmft=0  [59.4±12.09 months]  S-ECC: dmft= 9.3±3.6  [60.9± 8.8 months] | Unstimulated saliva (expectoration; morning; 1.5h fasting; 1.5h after toothbrushing) | Immuno-diffusion | IgG (mg/dL; mean ± sd):  Caries free = 8.49 ± 2.75;  S-ECC = 9.78 ± 3.26  **(p=0.046)** | GOOD |
| Hegde et al.  (2013**c**) | India | Caries-free (20)  [20-30 years-old]  Low caries (20)  [20-30 years-old]  Moderate caries (20)  [20-30 years-old]  High caries (20)  [20-30 years-old] | WHO | Caries-free: DMFT=0  Low caries: DMFT 1-5  Moderate caries: DMFT 6-10  High caries: DMFT>10 | Unstimulated saliva (drooling; morning; 1h fasting) | immunoglobulin estimation kit | IgG (mg/dL; mean ± sd):  Caries-free= 1357.11 ± 422.88  Low-caries= 1147.49 ± 422.88  Moderate caries= 1293.61 ± 422.88  High-caries= 1326.74 ± 422.88  **(p<0.001)**  *Calculated (mean±sd) for*  *Low-moderate-high caries = 1273.64±423.00* | FAIR |
| Razi et al.  (2020) | India | Caries-free (20)  [12-15 years-old]  Caries-active (20)  [12-15 years-old] | WHO | Caries-free: DMFS=0  Caries-active: DMFS≥10 | Unstimulated saliva (drooling; morning; 1.5h fasting) | single radial immune-diffusion method | IgG (mg/dL; mean ± sd):  Caries-free= 1.04±0.31  Caries-active= 0.87±0.14  (p=0.085) | GOOD |
