# Supplementary material for: Comparison of oral cavity protein abundance among caries-free and caries-affected individuals—a systematic review and meta-analysis
Source: Front Oral Health. 2023 Sep 15;4:1265817. doi: 10.3389/froh.2023.1265817 (PMC10540632; doi:10.3389/froh.2023.1265817)
Supplement: Supplementary file 5 [file Table5.docx]

**Table S5.** Concentration/activity of salivary antioxidant enzymes in the oral cavity of caries-free and caries-affected individuals

| **Study (year)** | **Country** | **Participants (n)**  **[Age; mean ± sd]** | **Criteria for caries diagnosis** | **Caries experience** | **Clinical sample** | **Method** | **Results** | **Quality** |
| --- | --- | --- | --- | --- | --- | --- | --- | --- |
| Lamberts et al.  (1984) | USA | Caries-free (29)  [19.3 ± 2.6 years-old]  Caries-active (29)  [20.4 ± 1.9 years-old] | WHO | Caries-free: DMFT = 0  Caries-active:  average= 10.2 carious lesions (range, 5-18) | Unstimulated saliva (drooling; morning] | ABTS method | Salivary Peroxidase (mU/mL; mean ± sd):  Caries-free: 123.9± 66.0  Caries-active: 168.3 ± 101.9  (p>0.05) | FAIR |
| Araújo et al.  (2020) | Brazil | Caries-free (30)  [17.8 ± 4.5 months-old]  Early carious lesions (30)  [23.6 ± 5:6 months-old]  Moderate carious lesions (30)  [32.6 ± 4.5 months-old]  Advanced carious lesions (30)  [35.0 ± 2.8 months-old] | ICCMS | Number of lesions:  Caries-free= 0  Early carious lesions= 1.93±0.94  Mod. carious lesions= 1.83±0.87  Adv. carious lesion= 2.57 ± 1.10 | Unstimulated saliva (cotton swab; morning; 2h fasting) | Maklund Method | Superoxide dismutase activity (UE/mL) by mg protein concentration:  Adv. Carious lesion group = mod. Carious lesion > early carious lesion = caries-free  **(p<0.001)**  A positive and strong correlation between caries severity and salivary SOD activity (Spearman’s r = 0.7320, **p < 0.0001**) | GOOD |
| Hegde et al.  (2014) | India | Caries-free (20)  [25-50 years-old]  Caries-active (60)  [25-50 years-old] | WHO | Caries-free: DMFT=0  Caries-active: DMFT>10 | Unstimulated saliva (2h fasting) | Nitro Blue Tetrazolium Chloride Reduction Method | Superoxide dismutase activity (U/mg protein; mean ± sd):  Caries-free=0.19 ± 0.049  Caries-active=0.35 ± 0.139  **(p<0.0001)** | FAIR |
| Silva et al.  (2016) | Brazil | Caries-free (30)  [0-3 years-old]  S-ECC (30)  [0-3 years-old] | WHO | Caries-free: dfms=0  S-ECC: dmfs= 3.7±3.44 | Unstimulated saliva (expectoration; morning; 2h fasting] | Maklund | Superoxide dismutase activity (UE/mL;  mean ± sd):  Caries-free= 26.8±9.09  S-ECC= 36.6±22.7  **(p<0.05)** | GOOD |
| Hegde et al.  (2013b) | India | Caries-free (20)  [25-50 years-old]  Low-caries (20)  [25-50 years-old]  Moderate-caries (20)  [25-50 years-old]  High-caries (20)  [25-50 years-old] | WHO | Low-caries: DMFT <3  Moderate caries: DMFT <10  High caries: DMFT >10 | Unstimulated saliva (noon; 2h fasting) | Myeloperoxidase activity was estimated based on  Matheston et al. (1981)  Glutathione peroxidase was estimated based on Rotruck  et al., 1973) | Myeloperoxidase activity  (pmol/dL; mean ± sd):  Caries-free= 7.73 ± 0.96  Low-caries= 23.46 ± 2.04  Moderate-caries= 42.69 ± 1.99  High-caries= 40.26 ± 3.11  **(p<0.0001)**  Glutathione peroxidase activity  (μg glutathione consumed/gprot/min;  mean ± sd):  Caries-free= 70.40 ± 21.90  Low-caries= 92.20 ± 29.14  Moderate-caries= 90.13 ± 33.62  High-caries= 90.14 ± 24.69  **(p=0.04)** | FAIR |

**Table S5 (cont).** Concentration/activity of salivary antioxidant enzymes in the oral cavity of caries-free and caries-affected individuals

| **Study (year)** | **Country** | **Participants (n)**  **[Age; mean ± sd]** | **Criteria for caries diagnosis** | **Caries experience** | **Clinical sample** | **Method** | **Results** | **Quality** |
| --- | --- | --- | --- | --- | --- | --- | --- | --- |
| Karthika et al. (2021) | India | Caries-free (50)  [10.21 ± 1.4 years-old]  Caries-active (50)  [10.21 ± 1.4 years-old] | WHO | Caries-free: DMFT=0  Caries-active: DMFT ≥5 | Unstimulated saliva (drooling) | Hydrogen peroxide method | Glutathione peroxidase activity  (nmol NAPDH oxidized/min/mL; mean ± sd):  Caries-free: 1.62 ± 0.14  Caries-active: 0.53 ± 0.08  **(p=0.001)** | FAIR |
| Mandel et al.  (1983) | A | Caries-resistant (27)  [from 24 to 40 years-old]  Caries-susceptible (13)  [from 24 to 40 years-old] | WHO | Caries-resistant: DMFS=0  Caries-susceptible: DFMS-=20 | Unstimulated saliva drooling) | Lactoperoxida-se was estimated based on Hydrogen peroxide and potassium iodide  Hypothiociani-te was estimated based on- 5-thio-2-nitro-benzoic acid (TNB) test  Thiocianate was estimated based on ferric nitrate method | Lactoperoxidase activity  (mg/dL; mean ± sd):  Parotid:  Caries-resistant: 0.24± 0.27  Caries-susceptible: 0.23± 0.15  (p>0.05)  Submandibular  Caries-resistant: 0.27 ± 0.25  Caries-susceptible: 0.16 ± 0.04  (p>0.05)  Hypothiocianite concentration  (µmol; mean ± sd):  Parotid:  Caries-resistant: 15.2 ± 8.7  Caries-susceptible: 21.0 ± 18.2  (p>0.05)  Submandibular  Caries-resistant: 24.0 ± 8.8  Caries-susceptible: 39.7 ± 25.8  (p>0.05)  Thiocianate concentration  (mg/dL; mean ± sd):  Parotid:  Caries-resistant: 5.1 ± 2.6  Caries-susceptible: 8.1 ± 5.5  (p>0.05)  Submandibular  Caries-resistant: 3.3 ± 2.6  Caries-susceptible: 6.7 ± 4.5  (p>0.05) | FAIR |
