# Supplementary material for: Comparison of oral cavity protein abundance among caries-free and caries-affected individuals—a systematic review and meta-analysis
Source: Front Oral Health. 2023 Sep 15;4:1265817. doi: 10.3389/froh.2023.1265817 (PMC10540632; doi:10.3389/froh.2023.1265817)
Supplement: Supplementary file 6 [file Table6.docx]

**Table S6.** Alpha-amylase concentration/activity in the oral cavity of caries-free and caries-affected individuals

| **Study (year)** | **Country** | **Participants (n)**  **[Age; mean ± sd]** | **Criteria for caries diagnosis** | **Caries experience** | **Clinical sample** | **Method** | **Results** | **Quality** |
| --- | --- | --- | --- | --- | --- | --- | --- | --- |
| Ahmad et al. (2021) | India | Caries-free (50)  [8-12 years old]  Caries-active (50)  [8-12 years-old] | WHO | Caries-free: DMFT or dmft=0  Caries-active: DMFT or dmft ≥ 5 | Unstimulated saliva  (drooling; morning; 1h fasting) | Hydrolysis of starch | Alpha-amylase activity (U/L; mean ± sd):  Caries-free= 83,530 ± 27,610  Caries-active= 68,420 ± 26,280  **(p<0.05)** | GOOD |
| Balekjian et al. (1975) | USA | Caries-resistant (11)  [17-21 years-old]  Caries susceptible (10)  [17-21 years-old] | WHO | Caries-susceptible: DMFT from 11 to 30; mean of 16.5 | Stimulated parotid saliva (cannulation) | Quantitative electrophoresis on polyacrylamide gel slabs (densitometer) | Alpha-amylase (mg/mL; mean ± sd)  Caries resistant=0.264±0.159  Caries susceptible=0.477 ±0.154  (p<0.1) | FAIR |
| Borghi et al.  (2016)* | Brazil | Caries-free (55)  [24-48 months-old]  Caries (45)  [24-48 months-old] | Nyvad (incl. non-cavitated lesions) | caries-free: dmfs and dmft=0  baseline caries-active:  dmfs= 7.88 ±9.98  dmft= 5.28 ± 4.64 | Unstimulated saliva (aspiration; morning; 1h fasting) | ELISA | Alpha-amylase activity (U/mL; medians and interquartile deviations):  Caries-free= 99.2±84  Caries= 55.6±52.8  **(p<0.0001)** | GOOD |
| de Farias; Bezerra (2003) | Brazil | Caries-free (20)  [39.5±7.12 months]  ECC (20)  [37.7±9.05 months) | WHO | Caries-free: dmfs=0  ECC: dfms= 16.4±8.9. | Unstimulated saliva (aspiration; morning; 1h fasting and 1h after toothbrusing) | alpha-amylase Colorimetric Test | Alpha-amylase activity (U/L; mean ± sd):  Caries-free= 71.90±48.60  ECC:= 122.80±101.50  (p>0.05) | FAIR |
| Doods et al.  (1997) | USA | Caries-free (38)  [mean 23.3 years-old]  Caries-active (49)  [mean 24.4 years-old] | NIDRC | Caries active: DMFS > 5 | Stimulated saliva – parotid (chemical) | SDS-PAGE  (densitometer)  HPLC | Alpha-amylase concentration  (µg/mL; mean±sem):  -Caries-free=214.9±24.8  -Caries-active=238.7±20.3  (p>0.05) | GOOD |
| Mojarad et al.  (2013) | Iran | Caries-free (84)  [3-6 years-old]  Caries-active (84)  [3-6 years-old] | Not informed | Not informed | Unstimulated saliva (spiiting; morning) | Spectrophotometric enzyme kinetics assay | Alpha-amylase activity (U/L; mean ± sd):  Caries-free= 0.042 ± 0.027  Caries-active=0.028 ± 0.021  **(p=0.001)** | GOOD |

**Table S6. cont.** Alpha-amylase concentration/activity in the oral cavity of caries-free and caries-affected individuals

| **Study (year)** | **Country** | **Participants (n)**  **[Age; mean ± sd]** | **Criteria for caries diagnosis** | **Caries experience** | **Clinical sample** | **Method** | **Results** | **Quality** |
| --- | --- | --- | --- | --- | --- | --- | --- | --- |
| Prabhakar et al. (2008) | India | Caries-free (20)  [9-10 years old]  Low-moderate caries (20)  [9-10 years old]  High-caries (20)  [9-10 years old] | WHO | Caries-free: DMFS+dfs=0  Low-moderate caries:  DMFS+dfs=3-8  High-caries: DMFS+dfs ≥ 9 | Stimulated (chewing; morning; 1h fasting and 1h w.o brushimg) | Maltotriose hydrolysis | Alpha-amylase activity (U/L; mean ± sd):  Caries-free= 85,000 ± 23,100.00  Low-moderate caries= 74,600 ± 21,300  High-caries= 64,600.00 ± 20,000  *Calculated (mean±sd) for low-moderate-high caries =69,600 ± 17,084.09* | FAIR |
| Singh et al.  (2015) | India | Caries-free (40)  [4 to 8 years-old]  Caries active (40)  [4 to 8 years-old] | WHO | caries-free DMFS =0  caries-active DMFS ≥ 5  (DMFS= 8.55±1.87) | Unstimulated saliva (drooling) | CNP-G3(2chloro-4- nitrophenyl-alpha-maltotrioside) method | Alpha-amylase activity (no unit provided):  Caries-free=7,809.10 ± 2,240.3  Caries active=19,793.4 ±14,651.0  **(p<0.05)** | FAIR |

*Cohort study but cross-sectional data reported
