# Supplementary material for: Comparison of oral cavity protein abundance among caries-free and caries-affected individuals—a systematic review and meta-analysis
Source: Front Oral Health. 2023 Sep 15;4:1265817. doi: 10.3389/froh.2023.1265817 (PMC10540632; doi:10.3389/froh.2023.1265817)
Supplement: Supplementary file 7 [file Table7.docx]

**Table S7.** Salivary enzymes (lysozyme, lactoferrin, protease and metalloprotease) concentration in the oral cavity of caries-free and caries-affected individuals

| **Study (year)** | **Country** | **Participants (n)**  **[Age; mean ± sd]** | **Criteria for caries diagnosis** | **Caries experience** | **Clinical sample** | **Method** | **Results** | **Quality** |
| --- | --- | --- | --- | --- | --- | --- | --- | --- |
| Felizardo et al.  (2010) | Brasil | Caries-free (27)  [12 years-old]  Dental caries (53)  [12 years-old] | WHO | Caries-free: DMFT=0  Dental caries: DMFT≥1 | Stimulated saliva  (afternoon; chewing; 2h fasting and 2h wo. toothbrusing) | SDS-PAGE | Lysozyme concentration (mg/mL):  Lysozyme was present in all of the participants in various concentrations. Twenty-four students showed salivary LZ concentrations below 81.55 mg/mL, 28 students showed concentrations between 81.56 mg/mL and 91.55 mg/mL and 28 students showed concentrations over 91.56 mg/mL. The Kruskall-Wallis test did not detect a relationship between lysozyme concentrations and the DMFT index (p=0.382) | GOOD |
| Lertsirivorakul et al. (2015) | Thailand | Caries-free (32)  [56.9 ± 7.2 months-old]  ECC (32)  [57.8 ± 8.2 months-old] | WHO | Caries-free: dmft=0  ECC: dmft= 12.5 ± 3.2  dmfs= 26.8 ± 15.5 | Unstimulated saliva (morning) | Western blotting | Lysozyme (intensity/mm^2^; mean±sd):  Caries-free= 6,781.9±2,128.6  ECC= 10,345.3±2,337.6  **(p<0.002)**  Lysozyme activity (µg/mL; mean±sd):  Caries-free= 16.3± 10.2  ECC= 24.4± 15.2  **(p=0.02)** | FAIR |
| Moslemi et al.  (2015) | Iran | Caries-free (21)  [from 36 to 71 months-old]  ECC (21)  [from 36 to 71 months-old] | WHO (incl. non-cavitated lesions) | Caries-free: dfms=0  ECC: dmfs ≥1 | Unstimulated saliva (morning; aspiration; 1h fasting) | ELISA | Lysozyme concentration (ng/mL; mean±sd):  Caries-free= 9,573.81 ± 1,148.3  ECC= 2,180 ± 653.52  **(p=0.04)** | GOOD |
| Stuchell; Mandel (1983) | Colombia | Caries-resistant (46)  [≥ 25 years-old]  Caries-susceptible (17)  [≥ 25 years-old] | Not informed | Caries-resistant: DMFS=0  Caries-susceptible: DMFS≥5 | Stimulated saliva (chemical) | Electro immuno-diffusion technique | Lysozyme concentration (mg/dL; mean±sd):  Parotid  Caries-resistant: 0.59 ± 0.62  Caries-susceptible: 0.55 ± 0.41  (p>0.05)  Submandibular-Sublingual  Caries-resistant: 2.1 ± 1.3  Caries-susceptible: 2.1 ± 1.6  (p>0.05) | FAIR |

**Table S7. (cont).** Salivary enzymes (lysozyme, lactoferrin, protease and metalloprotease) concentration in the oral cavity of caries-free and caries-affected individuals

| **Study (year)** | **Country** | **Participants (n)**  **[Age; mean ± sd]** | **Criteria for caries diagnosis** | **Caries experience** | **Clinical sample** | **Method** | **Results** | **Quality** |
| --- | --- | --- | --- | --- | --- | --- | --- | --- |
| Felizardo et al.  (2010) | Brasil | Caries-free (27)  [12 years-old]  Dental caries (53)  [12 years-old] | WHO | Caries-free: DMFT=0  Dental caries: DMFT≥1 | Stimulated saliva  (afternoon; chewing; 2h fasting and 2h wo. toothbrusing) | SDS-PAGE | Lactoferrin concentration (mg/mL):  Lactoferrin was absent (or not detected) in the saliva of 44 volunteers and was present in 36 participants. The Mann-Whitney test did not reveal a statistically significant relationship between salivary LF and the DMFT index (p=0.057) | GOOD |
| Moslemi et al.  (2015) | Iran | Caries-free (21)  [from 36 to 71 months-old]  ECC (21)  [from 36 to 71 months-old] | WHO (incl. non-cavitated lesions) | Caries-free: dfms=0  ECC: dmfs ≥1 | Unstimulated saliva (morning; aspiration; 1h fasting) | ELISA | Lactoferrin concentration (ng/mL; mean±sd):  Caries-free= 50.93 ± 24.44  ECC= 37.9 ± 16.43  (p=0.06) | GOOD |
| Hedenbjörk-Lager et al.  (2015) | Sweden | Caries-free (306)  [mean 50.6 ± 17.0 years-old]  Moderate caries (104)  [mean 50.6 ± 17.0 years-old]  High caries (41)  [mean 50.6 ± 17.0 years-old] | WHO | Caries-free: DS=0  Moderate caries: DS:1-2  High caries: DS≥3 | Stimulated saliva (chewing) | Immuno-flurometric assay | Metalloproteinase-8 concentration  (ng/mL; mean±sd)  Caries-free= 254.6±189.8 (n=305)  Moderate caries= 345.0±371.3 (n=104)  High caries= 426.7±355.4 (n=40)  **(p<0.001)** | FAIR |
| Yang et al.  (2015b) | China | Caries-free (46)  [6 years-old]  Low-dental caries (49)  [6 years-old]  High dental caries (33)  [6 years-old] | WHO | Caries-free:  DMFT=0  Low dental caries:  DMFT= 1.98±0.83  High dental caries:  DMFT= 6.33±2.76 | Unstimulated saliva (morning; drooling; 12h fasting) | ELISA | Proteinase-3 concentration  (ng/mL; mean±sd):  Caries-free= 17.82 ±7.31  Low dental caries= 12.7 ±6.19*  High dental caries= 11.07 ±7.10*  **(*p<0.05 compared with caries-free)** | FAIR |
