# Supplementary material for: Comparison of oral cavity protein abundance among caries-free and caries-affected individuals—a systematic review and meta-analysis
Source: Front Oral Health. 2023 Sep 15;4:1265817. doi: 10.3389/froh.2023.1265817 (PMC10540632; doi:10.3389/froh.2023.1265817)
Supplement: Supplementary file 8 [file Table8.docx]

**Table S8.** Carbonic anhydrase VI activity/concentration in the oral cavity of caries-free and caries-affected individuals

| **Study (year)** | **Country** | **Participants (n)**  **[Age; mean ± sd]** | **Criteria for caries diagnosis** | **Caries experience** | **Clinical sample** | **Method** | **Results** | **Quality** |
| --- | --- | --- | --- | --- | --- | --- | --- | --- |
| Borghi et al.  (2016) | Brazil | Caries-free (55)  [from 24 to 48 months-old]  Caries (45)  [from 24 to 48 months-old] | Nyvad (incl. non-cavitated lesions) | caries-free: dmfs and dmft=0  baseline caries-active:  dmfs= 7.88 ±9.98  dmft= 5.28 ± 4.64 | Unstimulated saliva (aspiration; morning; 1h fasting) | Zymophaphy | Carbonic anhydrase VI activity  (pixel area):  (medians and interquartile deviations):  Caries-free= 0.25±0.43  Caries= 0.31±0.65  **(p<0.05)** | GOOD |
| Frasseto et al.  (2012) | Brazil | Caries-free (13)  [57.2 ± 7.2 months-old]  Caries (17)  [62.8 ± 17.5 months-old] | WHO (incl. non-cavitated lesion) | Not informed | Stimulated saliva (chewing; morning; 0.5h fasting) | Zymography | Carbonic anhydrase VI activity  (pixel area) (mean ± sd):  Caries-free=19,130.79±16,911.68  Caries=42,752.11±32,476.62  (p>0.05) | FAIR |
| Öztürk et al.  (2008) | Turkey | Caries-free (16)  [from 19 to 25 years-old]  Caries (21)  [from 19 to 25 years-old] | WHO | Caries-free: DMFT = 0  Caries: DMFT= 5.6 | Unstimulated saliva (expectoration; morning; 12h fasting) | method of Verpoorte et al. (1967) | Carbonic anhydrase VI activity  (U/g protein; mean ± sd):  Caries-free= 26.8 ± 6.8  Caries= 27.2 ± 11.03  (p>0.05) | FAIR |
| Picco et al.  (2017) | Brazil | Caries-free (37)  [from 7 to 9 years-old]  Caries (37)  [from 7 to 9 years-old] | WHO (incl. non-cavitated lesions) | Caries: DMFT=3.162 | Stimulated saliva (chewing; 1h fasting) | Zymography (activity)  ELISA  (concentration) | Carbonic anhydrase VI activity  (pixel area; mean ± sd):  Caries-free=1.383±1,076  Caries=3.391 ± 2.046  **(p<0.0001)**  Carbonic anhydrase VI concentration  (ng/uL; mean ± sd):  Caries-free= 0.8561±0.7141  Caries= 0.4255±0.3835  **(p=0.0006)** | FAIR |
| Picco et al.  (2019) | Brazil | Caries-free (33)  [from 7 to 9 years-old]  Caries (34)  [from 7 to 9 years-old] | WHO | Caries-free: DMFT=0  Caries: DMFT= 3.162 ±1.385 | Supragingival biofilm (pool from buccal surfaces; 48h wo. Toothbrushing) | Zymography  (activity)  ELISA  (concentration) | Carbonic anhydrase VI activity  (pixel area; mean ± sd):  Caries-free= 17.65±9.52  Caries= 25.96±16.41  **(p=0.0421)**  Carbonic anhydrase VI concentration  (ng/uL; mean ± sd):  Caries-free= 3.507±4.014  Caries= 1.693±1.802  **(p=0.0335)** | FAIR |

**Table S8 (cont).** Carbonic anhydrase VI activity/concentration in the oral cavity of caries-free and caries-affected individuals

| **Study (year)** | **Country** | **Participants (n)**  **[Age; mean ± sd]** | **Criteria for caries diagnosis** | **Caries experience** | **Clinical sample** | **Method** | **Results** | **Quality** |
| --- | --- | --- | --- | --- | --- | --- | --- | --- |
| Sousa et al.  (2020) | Brazil | Caries-free (22)  [mean 4.55±0.60 years-old]  ECC (22)  [mean 4.59±0.50 years-old] | WHO (incl. non-cavitated lesion) | ECC: dmfs=12.23±7.98 | Supragingival biofilm (pool from buccal surfaces; morning; 1h fasting and 48h wo. Toothbrushing)  Stimulated saliva (chewing; morning; 1h fasting) | Zymography | Carbonic anhydrase IV activity  (pixel/mg of total protein):  Supragingival plaque:  Caries-free= 2.4±1.69  ECC= 4.2±2.84  **(p<0.05)**  Saliva:  Caries-free= 4.0±2.0  ECC= 5.3±1.84  **(p<0.05)** | GOOD |
| Szabó  (1974) | Hungary | Caries-free (8)  [from 7 to 14 years-old]  Caries-active (8)  [from 7 to 14 years-old] | Not informed  (WHO?) | caries-free= DMFT =0  Caries active= DT ≥ 10 | Unstimulated saliva | method of Krebs and Roughton (1948) modified by Gloster (1955) | Carbonic anhydrase IV activity  (U/mL; mean ± sd):  Caries-free= 2.96 ±0.97  Caries active= 1.28±0.25  (p<0.2) | POOR |
