# Supplementary material for: Comparison of oral cavity protein abundance among caries-free and caries-affected individuals—a systematic review and meta-analysis
Source: Front Oral Health. 2023 Sep 15;4:1265817. doi: 10.3389/froh.2023.1265817 (PMC10540632; doi:10.3389/froh.2023.1265817)
Supplement: Supplementary file 9 [file Table9.docx]

**Table S9.** Urease and ADS activities in the oral cavity of caries-free and caries-affected individuals

| **Study (year)** | **Country** | **Participants (n)**  **[Age; mean ± sd]** | **Criteria for caries diagnosis** | **Caries experience** | **Clinical sample** | **Method** | **Results** | **Quality** |
| --- | --- | --- | --- | --- | --- | --- | --- | --- |
| Moncada et al.  (2015) | Chile | Caries-free (15)  [8 year-old]  Moderate caries (31)  [8 year-old]  High caries (19)  [8 year-old] | WHO | Caries-free: dmft=0  Moderate caries: dt≥3 (DMFT= 3.09) (at least 3 teeth with enamel caries)  High caries: dt≥4; (DMFT=5.84) (at least 4 teeth with dentinal caries | Unstimulated saliva (drooling; 12h wo. toothbrushing) | Production of ammonia (spectrophotometry) | Urease activity (ammonia production)  (μmol x min^-1^/ mg protein; mean±sd):  Caries-free= 0.69±0.7  Moderate caries= 0.45±0.43  High caries= 0.39±0.55  **(p=0.048)**  *Calculated means for caries: 0.42 ± 0.47* | GOOD |
| Nascimento et al.  (2009) | USA | Caries-free (13)  [mean age 33 years-old)  Caries-active (21)  [mean age 33 years-old)  Caries experienced (11)  [mean age 33 years-old) | WHO | Caries-free: DMFT=0  Caries-active: DT ≥ 4; MFT ≥ 0  Caries-experienced= DT=0; MFT > 0 | Supragingival biofilm (pool of smooth dental surfaces of incisors and molar regions; morning; 16h wo. toothbrushing)  Unstimulated saliva (expectoration; morning) | Production of ammonia (spectrophotometry) | Urease activity (ammonia production)  (μmol x min^-1^/ mg protein; mean±sd):  Supragingival biofilm  Caries-free= 4.13±0.77  Caries-active= 1.51±0.60  Caries-experienced= 3.22±0.84  **(p=0.014)**  Saliva  Caries-free= 0.60±0.39  Caries-active= 0.52±0.31  Caries-experienced= 1.3±0.42  (p>0.05) | FAIR |
| Reyes et al.  (2014) | Chile | Caries-free (10)  [>18 years-old]  Caries-active (13)  [>18 years-old] | WHO | Caries-free: DMFT=0  Caries-active: DMFT≥4 | Supragingival biofilm (pool of buccal surfaces of upper and lower teeth; morning 12h wo. toothbrushing)  Unstimulated saliva (expectoration; morning 12h fasting) | Production of ammonia (spectrophotometry) | Urease activity (ammonia production)  (μmol x min^-1^/ mg protein; mean±sd):  Supragingival biofilm  Caries-free= 18.12±26.929  Caries-active= 0.37 ±0.156  **(p=0.033)**  Saliva  Caries-free= 3.024 ± 2.791  Caries-active= 0.437 ± 0.196  **(p=0.010)** | GOOD |

**Table S9 (cont).** Urease and ADS activities in the oral cavity of caries-free and caries-affected individuals

| **Study (year)** | **Country** | **Participants (n)**  **[Age; mean ± sd]** | **Criteria for caries diagnosis** | **Caries experience** | **Clinical sample** | **Method** | **Results** | **Quality** |
| --- | --- | --- | --- | --- | --- | --- | --- | --- |
| Shu et al.  (2007) | USA | Caries-free (25)  [24.9 years-old)  Caries active (8)  [26.6 years-old) | WHO | Caries-free= DMFT=0  Caries active=DT≥6 | Supragingival biofilm (pool of surfaces of one-half of mouth, except lingual surfaces of lower anterior teeth; morning 16h wo. toothbrushing)  Unstimulated saliva (expectoration; morning 16h fasting) | Production of ammonia (spectrophotometry) | Urease activity (ammonia production)  (μmol x min^-1^/ mg protein; mean±sd):  Supragingival biofilm  Caries-free= 3.11± 3.25  Caries-active= 1.0± 0.65  **(p<0.0001)**  Saliva  Caries-free= 0.41±0.28  Caries-active= 0.59±0.69  **(**p=0.81) | FAIR |
| Moncada et al.  (2015) | Chile | Caries-free (15)  [8 year-old]  Moderate caries (31)  [8 year-old]  High caries (19)  [8 year-old] | WHO | Caries-free: dmft=0  Moderate caries: dt≥3 (DMFT= 3.09) (at least 3 teeth with enamel caries)  High caries: dt≥4; (DMFT=5.84) (at least 4 teeth with dentinal caries | Unstimulated saliva (drooling; 12h wo. toothbrushing) | Production of ammonia (spectrophotometry) | ADS activity (ammonia production)  (μmol x min^-1^/ mg protein; mean±sd):  Caries-free= 2.53±1.42  Moderate caries= 2.31±1.57  High caries= 1.97±2.0  (p=0.162)  *Calculated means for caries: 2.14±1.73* | GOOD |
| Nascimento et al.  (2009) | USA | Caries-free (13)  [mean age 33 years-old)  Caries-active (21)  [mean age 33 years-old)  Caries experienced (11)  [mean age 33 years-old) | WHO | Caries-free: DMFT=0  Caries-active: DT ≥ 4; MFT ≥ 0  Caries-experienced= DT=0; MFT > 0 | Supragingival biofilm (pool of smooth dental surfaces of incisors and molar regions; morning; 16h wo. toothbrushing)  Unstimulated saliva (expectoration; morning) | Production of ammonia (spectrophotometry) | ADS activity (ammonia production)  (μmol x min^-1^/ mg protein; mean±sd):  Supragingival biofilm  Caries-free= 1.36±0.25  Caries-active= 0.74±0.20  Caries-experienced=1.41±0.28  **(**p>0.05)  Saliva  Caries-free= 1.08 ± 0.17  Caries-active= 0.21 ± 0.13  Caries-experienced=0.69 ± 0.18  **(p=0.004)** | FAIR |

**Table S9 (cont).** Urease and ADS activities in the oral cavity of caries-free and caries-affected individuals

| **Study (year)** | **Country** | **Participants (n)**  **[Age; mean ± sd]** | **Criteria for caries diagnosis** | **Caries experience** | **Clinical sample** | **Method** | **Results** | **Quality** |
| --- | --- | --- | --- | --- | --- | --- | --- | --- |
| Nascimento et al.  (2013) | USA | Caries-free (52)  [mean 7.8±3.6 years-old]  Caries-active (38)  [mean 7.8±3.6 years-old]  Caries-experienced (10)  [mean 7.8±3.6 years-old] | ICDAS | Caries-free: DMFT=0  Caries-active: DT ≥ 2; MFT ≥ 0  Caries-experienced= DT=0; MFT > 0 | Supragingival biofilm (individually collected from caries free, enamel caries and dentinal caries surfaces; morning; 8h wo. toothbrushing)  Unstimulated saliva (expectoration; morning) | citrulline production from arginine | ADS activity  (citrulline production from arginine)  (nmol x min^-1^/ mg protein; mean±sd):  Supragingival plaque  Caries-free= 377.1±252.6  Caries-active= 291.7 ±233.3  Caries-experienced= 299.3±230.6  (p>0.05)  Saliva:  no differences were observed in saliva ADS activity among the groups. | GOOD |
| Reyes et al.  (2014) | Chile | Caries-free (10)  [>18 years-old]  Caries-active (13)  [>18 years-old] | WHO | Caries-free: DMFT=0)  Caries-active: DMFT≥4 | Supragingival biofilm (pool of buccal surfaces of upper and lower teeth; morning; 12h wo. toothbrusing))  Unstimulated saliva (expectoration; morning 12h fasting) | Production of ammonia (spectrophotometry) | ADS activity (ammonia production)  (μmol x min^-1^/ mg protein; mean±sd):  Supragingival biofilm  Caries-free= 8.83±10.666  Caries-active= 1.21±0.392  **(p=0.026)**  Saliva  Caries-free= 6.0 ±5.808  Caries-active= 1.35±0.548  **(p=0.020)** | GOOD |
| VanWuyckhuysel et al. (1995) | USA | Caries-free (20)  [from 50 to 83 years-old]  Caries-susceptible adults (19)  [from 50 to 74 years-old] | WHO | Caries-free adults:  median DMFS=0;  Caries susceptible adults:  median DMFS=48  (range= 23 to 125) | Stimulated parotid saliva (chemical; morning; 2h fasting) | Hewlett  Packard Amino Quant II amino acid analyzer | Arginine concentration  [median (range), nM/mL]  Caries-free adults: 14.61 (8.98-42.79);  Caries-susceptible adults:  10.85 (8.63-15.84)  **(p≤0.0025)**  Lysine concentration  [median (range), nM/mL]  Caries-free adults: 2.44 (0-5.02);  Caries-susceptible adults:  0.66 (0-4.10)  **(p≤0.0025)** | FAIR |
