# Supplementary material for: Comparison of oral cavity protein abundance among caries-free and caries-affected individuals—a systematic review and meta-analysis
Source: Front Oral Health. 2023 Sep 15;4:1265817. doi: 10.3389/froh.2023.1265817 (PMC10540632; doi:10.3389/froh.2023.1265817)
Supplement: Supplementary file 10 [file Table10.docx]

**Table S10.** Salivary concentration of albumin, cystatins, histatins, mucin, proline-rich proteins and statherin in the oral cavity of caries-free and caries-affected individuals

| **Study (year)** | **Country** | **Participants (n)**  **[Age; mean ± sd]** | **Criteria for caries diagnosis** | **Caries experience** | **Clinical sample** | **Method** | **Results** | **Quality** |
| --- | --- | --- | --- | --- | --- | --- | --- | --- |
| Bachtiar et al.  (2018) | Indonesia | Caries-free (16)  [from 3 to 5 years-old]  ECC (16)  [from 3 to 5 years-old] | Not informed | Not informed | Unstimulated saliva (aspiration) | SDS-PAGE | Protein band:  Five protein band were found **more frequently in caries-free:**  *15 kDa (suspected as cystatin)  *25 kDa (suspected as basic PRPs (bPRPs)  *60 kDa (suspected as α-amylase)  *65 kDa (suspected as serum albumin)  *95 kDa (suspected as the secretory component, which are the transporters of IgA in epithelial cells) | POOR |
| Bhalla et al.  (2010) | India | Caries-free (50)  [from 4 to 6 years-old]  ECC (50)  [from 4 to 6 years-old] | WHO | Not informed | Unstimulated saliva (expectoration; morning) | SDS-PAGE | Protein band:  Caries-free: higher number of proline-rich protein bands  ECC: higher number of glycoprotein bands | FAIR |
| Balekjian et al. (1975) | USA | Caries-resistant (11)  [from 17 to 21 years-old]  Caries susceptible (10)  [from 17 to 21 years-old] | WHO | Caries-susceptible: DMFT from 11 to 30; mean of 16.5 | Stimulated parotid saliva (cannulation) | Electrophoresis (densitometry) | Proteins (mg/mg total protein; mean±sd)  *Anode protein (positive charged; ex. alpha-amylase)  Caries-resistant= 0.566±0.096  Caries-susceptible= 0.681±0.073  **(p<0.01)**  *Cathode protein (negative charged; ex. Lactoperoxidase and lisozyme)  Caries-resistant= 0.439±0.099  Caries-susceptible= 0.319±0.073  **(p<0.01)** | FAIR |

**Table S10 (cont).** Salivary concentration of albumin, cystatins, histatins, mucin, proline-rich proteins and sthaterin in the oral cavity of caries-free and caries-affected Individuals

| **Study (year)** | **Country** | **Participants (n)**  **[Age; mean ± sd]** | **Criteria for caries diagnosis** | **Caries experience** | **Clinical sample** | **Method** | **Results** | **Quality** |
| --- | --- | --- | --- | --- | --- | --- | --- | --- |
| Doods et al.  (1997) | USA | Caries-free (38)  [mean 23.3 years-old]  Caries-active (49)  [mean 24.4 years-old] | NIDRC | Caries active: DMFS > 5 | Stimulated saliva – parotid (chemical) | SDS-PAGE  (densitometer)  HPLC | Protein concentration (mean±sem):  *Histatins (µg/mL)  -Caries-free=67.6±8.5  -Caries-active=64.8±7.8  (p>0.05)  *Statherin(area/mL)  -Caries-free= 26.1±3.0  -Caries-active= 28.1±2.4  (p>0.05)  *Acidic PRP (area/mL)  -Caries-free= 201.8±16.5  -Caries-active= 254.1±19.9  (p>0.05)  *Basic PRP area/mL)  -Caries-free= 679.4±72.3  -Caries-active=734.2±57.9  (p>0.05) | GOOD |
| Szkaradkiewcz-Karpinska et al. (2019) | Poland | Caries free (19)  [mean 29.42±3.71 years-old]  Severe caries (26)  [mean 32±5.74 years-old] | WHO | Caries free: DMFT=0  Severe caries: DMFT=16.11±2.26 | Unstimulated saliva (expectoration; morning; 2h fasting) | ELISA | Mucin level (ng/mL; mean±sd)  MUC5B:  *Caries-free= 0.63 ± 0.35  Severe caries= 0.38 ± 0.32  **(p= 0.023)**  MUC7:  *Caries-free= 5.47 ± 1.18  Severe caries= 1.39 ± 0.86  **(p< 0.0001)** | GOOD |

**Table S10 (cont).** Salivary concentration of albumin, cystatins, histatins, mucin, proline-rich proteins and sthaterin in the oral cavity of caries-free and caries-affected individuals

| **Study (year)** | **Country** | **Participants (n)**  **[Age; mean ± sd]** | **Criteria for caries diagnosis** | **Caries experience** | **Clinical sample** | **Method** | **Results** | **Quality** |
| --- | --- | --- | --- | --- | --- | --- | --- | --- |
| Koopaie et al. (2021) | Iran | Caries-free (20)  [mean 63.25 ± 8.32 months]  ECC (20)  [mean 63.70 ± 8.32 months] | WHO (incl. non-cavitated lesions) | Not informed | Unstimulated saliva (suction; morning; 30 min fasting) | ELISA | Cystatin-S (ng/mL; mean ± sd):  Caries-free: 370.06 ± 128.87  ECC: 191.55 ± 81.90  **(p=0.032)** | FAIR |
| Nireeksha et al.  (2017) | India | Caries-free (20)  [25-40 years-old]  Low caries (20)  [25-40 years-old]  Moderate caries (20)  [25-40 years-old]  High caries (20)  [25-40 years-old] | WHO | Caries-free: DMFT=0  Low caries: DMFT=1-3  Moderate caries: DMFT=4-10  High-caries: DMFT>10 | Unstimulated saliva (drooling; morning; 2h fasting) | Alcian blue | Mucin (mg/dL; mean ± sd):  Caries-free= 1.19 ± 0.20  Low-caries= 1.20 ± 0.151  Moderate caries= 1.69 ± 0.179  High-caries= 2.05±0.240  **(p<0.001)**  *Calculated (mean±sd) for*  *Low-moderate-high caries = 1.74±0.4* | FAIR |
| Roa et al.  (2008) | Colombia | Caries-free (49)  [> 18 years-old]  History of caries (49)  [> 18 years-old]  Caries active (47)  [> 18 years-old] | WHO | History of caries: with amalgam or resin fillings and currently free of  caries  Caries active: with multiple cavities, including enamel and dentine. | Unstimulated saliva (drooling; 2h fasting) | SDS-PAGE | Protein (electrophoresis)  The correlation between clinical diagnosis to the electrophoretic patterns of salivary proteins showed no statistically significant difference among the studied group (p=0.3893)  17 kDa protein was more frequent in men with active caries **(p=0.037)** | FAIR |

**Table S10 (cont).** Salivary concentration of albumin, cystatins, histatins, mucin, proline-rich proteins and sthaterin in the oral cavity of caries-free and caries-affected individuals

| **Study (year)** | **Country** | **Participants (n)**  **[Age; mean ± sd]** | **Criteria for caries diagnosis** | **Caries experience** | **Clinical sample** | **Method** | **Results** | **Quality** |
| --- | --- | --- | --- | --- | --- | --- | --- | --- |
| Szkaradkiewcz-Karpinska et al. (2017) | Poland | Caries-free (18)  [20-26 years-old]  Very low caries (20)  [20-32 years-old]  Low caries (20)  [21-35 years-old]  Moderate caries (24)  [20-35 years-old]  High caries (24)  [20-35 years-old] | WHO | Caries-free: DMFT=0  Very low caries: DMFT= 2.3 ± 1.0  Low caries: DMFT= 6.2 ± 1.3  Moderate caries: DMFT= 10.9 ± 1.8  High caries: DMFT= 19.5 ± 3.5 | Unstimulated saliva (expectoration; morning) | ELISA | Acidic PRP 1/2 concentration (APRPs) (ng/mL; mean ± sd):  Caries-free: 15.2 ± 2.6a  Very low caries: 15.9 ± 2.1a  Low caries: 18.6 ± 3.2b  Moderate caries: 35.4 ± 4.6c  High caries: 39.8 ± 5.1d  The obtained values of APRP-1/2 in group low, moderate, and high caries were significantly higher than results obtained in caries-free (**p<0.05**; Mann-Whitney test). | FAIR |
| Vieira et al.  (2020) | USA | Caries-free (20)  [mean 9.15 years-old]  Caries-active (71)  [mean 13.53 years-old] | Not clearly informed | Caries free: DMFT=0; DMFS=0  Caries active:  DMFT (Mean and Range)  CA: 4.82 (1-17)  DMFS (Mean and Range)  CA: 8.9 (1-41)  Surfaces were defined as decayed if the carious lesions extended into dentine or were circumscribed to the dental enamel and appeared active (white spot lesions) | Unstimulated saliva | Eletrophoresis | Histatin was more likely to be non-detectable or reduced in caries-free children (OR = 7.56; 95% CI 1.62-35.13)  No differences were found for amylase and agglutinin | FAIR |
| Zengo et al.  (1971) | USA | Caries resistant (20)  [adults]  Caries susceptible (20)  [adults] | Not informed | Caries-free: DF=0  Caries susceptible: DMF≥15 | Stimulated saliva  (citric acid; morning; 2h fasting) | radial immune-diffusion method | Albumin (mg/dL; mean ± sd):  Stimulated parotid saliva:  Caries-resistant: 0.7 ± 0.5  Caries-susceptible: 0.6 ± 0.2  (p>0.05)  Stimulated submaxillary saliva:  Caries-resistant: 1.10 ±0.80  Caries-susceptible: 0.3 ± 0.2  **(p<0.05)** | FAIR |
