# Supplementary material for: Comparison of oral cavity protein abundance among caries-free and caries-affected individuals—a systematic review and meta-analysis
Source: Front Oral Health. 2023 Sep 15;4:1265817. doi: 10.3389/froh.2023.1265817 (PMC10540632; doi:10.3389/froh.2023.1265817)
Supplement: Supplementary file 11 [file Table11.docx]

**Table S11.** Salivary antimicrobial peptides concentration in the oral cavity of caries-free and caries-affected individuals

| **Study (year)** | **Country** | **Participants (n)**  **[Age; mean ± sd]** | **Criteria for caries diagnosis** | **Caries experience** | **Clinical sample** | **Method** | **Results** | **Quality** |
| --- | --- | --- | --- | --- | --- | --- | --- | --- |
| Al-Ali et al  (2021) | Iraq | Caries-free (44)  [from 6-7 years-old]  Caries (moderate/severe)(44)  [from 6-7 years-old] | ICDAS | Caries-free: ICDAS d=0 and dmf=0 Caries: showed different stages of caries severity (d3-d6) with highest percentage 63.64% having severe extensive caries (d6). | Unstimulated saliva (expectoration; 1h fasting) | ELISA | LL-37 (ng/mL; mean±sd):  Caries-free= 2.179 ± 1.883  Caries= 1.83 ± 1.861  (p=0.384)  hBD (ug/mL; mean±sd):  Caries-free= 275.352 ± 256.56  Caries= 176.495 ± 175.932  **(p=0.038)** | FAIR |
| Barrera et al.  (2013) | Venezuela | Caries-free (44)  [from 18 to 32 years-old]  Mild caries (19)  [from 18 to 32 years-old]  Moderate caries (25)  [from 18 to 32 years-old]  Severe caries (12)  [from 18 to 32 years-old] | Not clearly informed | Caries-free: score=0  Mild caries: score=1  Moderate caries: score=2  Severe caries: score≥3 | Unstimulated saliva (drooling) | ELISA | hBD-2 (μg/ml; median):  Caries-free=1.88  Caries= 7.26  **(p<0.05)**  hBD-4 (μg/ml; median):  Caries-free= 0.86  Caries= 4.25  **(p<0.05)**  hNP-4 (μg/ml; median):  Caries-free= 3.91  Caries= 4.52  (p=0.05) | FAIR |
| Colombo et al.  (2016b) | Brazil | Caries-free (29)  [mean 48.3±8.5 months-old];  ECC (25)  [mean 49.6±7.7 months-old]  S-ECC (29)  [mean 50.2 ±9.9 months-old) | AAPD (incl. non-cavitated lesion) | Caries-free: Dmfs= 0±0  ECC: 2.36±0.95;  S-ECC: 19.34±14.97  dmfs + white spot  ECC: 2.76 ±1.23;  S-ECC: 24.41±17.94 | Unstimulated saliva (expectoration; 1h fasting) | ELISA | LL-37 (ng/mL)  hBD-2 (ng/mL)  hBD-3 (ng/mL)  HTN-5 (ng/mL)  No difference among groups | FAIR |
| Davidopoulou et al.  (2012) | Greece | Caries-free (28)  [mean age 8.7 years-old]  Low/moder. Activity (9)  [mean age 8.7 years-old]  High caries activity (10)  [mean age 8.7 years-old] | WHO | Caries free: DT=0  Low/moder.activity: DT= 1–3  High caries activity: DT ≥4). | Unstimulated saliva (expectoration; afternoon) | ELISA | LL-37 (ng/mL; median; min-max):  Caries-free= 24.00; 0.22–165.00  Low/Moder.caries activity=  26.00; 1.15–275.00  High caries activity= 7.50; 0.75–50.00  High caries activity showed the lowest LL-37 concentration **(p<0.05)** | GOOD |

**Table S11 (cont).** Salivary antimicrobial peptides concentration in the oral cavity of caries-free and caries-affected individuals

| **Study (year)** | **Country** | **Participants (n)**  **[Age; mean ± sd]** | **Criteria for caries diagnosis** | **Caries experience** | **Clinical sample** | **Method** | **Results** | **Quality** |
| --- | --- | --- | --- | --- | --- | --- | --- | --- |
| Jurczak et al. (2015) | Poland | Caries-free (41)  [mean age 5 ± 1.5 years-old]  ECC (41)  [mean age 5 ± 2.3 years-old] | ICDAS | ECC:  ICDAS II: 1-2 (n=17)  ICDAS II: ≥3 (n=24) | Unstimulated saliva (expectoration; morning; fasting) | ELISA | HST-5 (ng/mL; mean±sd):  Caries-free: 15.29 ± 1.16  ECC: 50.75 ± 2.11  **(p=0.0002)**  hBD-2 (ug/mL; mean±sd):  Caries-free: 0.00215 ± 0.00007  ECC: 0.00229 ± 0.00005  **(p=0.0417)** | FAIR |
| Luthfi et al.  (2019) | Indonesia | Caries-free (20)  [4-6 years-old]  ECC (20)  [4-6 years-old] | Not informed | Caries‑free= dmft = 0  ECC=dmft ≥ 6 | Salivary oral rinse | ELISA | HNP1-3 (ug/mL; mean±sd):  Caries-free= 0.00014±0.00003  ECC=0.00017 ±0.00004  **(p= 0.009)** | FAIR |
| Phattarataratip et al. (2011)* | USA | Caries-free (30)  [13 years-old]  Caries-active (30)  [13 years-old] | WHO | Caries-active: 3 or more carious or filled surfaces (D2-3F). | Stimulated saliva (chewing) | ELISA | HNP1-3(ug/mL; mean±sd):  Caries-free= 1.779±1.055  Caries-active= 2.047±1.254  (p=0.37)  LL-37(ng/mL; mean±sd):  Caries-free= 15.70±12.86  Caries-active= 15.92±7.50  (p=0.94)  hDB-2(ug/mL; mean±sd):  Caries-free= 0.00230 ± 0.00246  Caries-active= 0.00216 ± 0.00191  (p=0.80)  hDB-3(ug/mL; mean±sd):  Caries-free= 0.00085±0.00083  Caries-active= 0.00086±0.00090  (p=0.23) | FAIR |
| Tao et al.  (2005) | USA | Caries-free (51)  [11-15 years-old]  Caries (92)  [11-15 years-old] | WHO | Caries= caries score>1 | Unstimulated saliva (morning) | ELISA | HNP1-3 (ug/mL; median, interquartile range):  Caries-free=0.89 (0.24 to 0.9)  Caries= 0.50 (0.24 to 0.9)  **(p=0.004)** | FAIR |

**Table S11 (cont).** Salivary antimicrobial peptides concentration in the oral cavity of caries-free and caries-affected individuals

| **Study (year)** | **Country** | **Participants (n)**  **[Age; mean ± sd]** | **Criteria for caries diagnosis** | **Caries experience** | **Clinical sample** | **Method** | **Results** | **Quality** |
| --- | --- | --- | --- | --- | --- | --- | --- | --- |
| Toomarian et al. (2011) | Iran | Caries-free (27)  [from 3-5 years-old]  Moderate caries (30)  [from 3-5 years-old]  S-ECC (30)  [from 3-5 years-old] | WHO | Not informed | Unstimulated saliva (aspiration; morning) | ELISA | HNP1-3(ug/mL; mean±sd):  Caries-free= 0.08413 ± 0.01734  Moderate caries= 0.08691 ± 0.01361  S-ECC= 0.08707±0.00779  (p>0.05)  *Calculated (mean±sd) for moderate-S-ECC = 0.08695 ± 0.0836* | FAIR |

*Cohort study but cross-sectional data reported
