# Supplementary material for: Comparison of oral cavity protein abundance among caries-free and caries-affected individuals—a systematic review and meta-analysis
Source: Front Oral Health. 2023 Sep 15;4:1265817. doi: 10.3389/froh.2023.1265817 (PMC10540632; doi:10.3389/froh.2023.1265817)
Supplement: Supplementary file 12 [file Table12.docx]

**Table S12.** Quality assessment of cross-sectional studies

| **Study (year)** | **Q1** | **Q2** | **Q3** | **Q4** | **Q5** | **Q6** | **Q7** | **Q8** | **Q9** | **Q10** | **Overall** |
| --- | --- | --- | --- | --- | --- | --- | --- | --- | --- | --- | --- |
| Ahmad et al. (2021) | YES | YES | YES | NO | YES | YES | YES | NR | YES | YES | GOOD |
| Ahmadi-Motamayel et al. (2013a) | YES | YES | YES | NO | YES | NO | YES | NR | YES | YES | FAIR |
| Ahmadi-Motamayel et al. (2013b) | YES | YES | YES | NO | YES | NO | YES | NR | YES | YES | FAIR |
| Ahmadi-Motamayel et al. (2018) | YES | YES | YES | YES | YES | YES | YES | NR | YES | YES | GOOD |
| Al-Ali et al. (2021) | NO | YES | YES | NO | YES | YES | YES | NR | YES | YES | FAIR |
| Al- Amoudi et al. (2007) | YES | YES | YES | NO | YES | YES | YES | NR | YES | YES | GOOD |
| Aliakbarpour et al. (2021) | YES | YES | YES | NO | NO | NO | YES | NR | YES | NO | FAIR |
| Araújo et al.(2020) | YES | YES | YES | NO | YES | YES | YES | NR | YES | YES | GOOD |
| Bachtiar et al. (2018) | YES | NO | NR | NO | YES | NR | YES | NR | YES | NO | POOR |
| Bagherian et al. (2008) | YES | YES | YES | NO | YES | YES | YES | NR | YES | YES | GOOD |
| Balekjian et al. (1975) | YES | YES | NO | NO | YES | NO | YES | NR | YES | NO | FAIR |
| Banda et al. (2017) | YES | YES | YES | NO | YES | YES | YES | NR | YES | YES | GOOD |
| Barrera et al. (2013) | YES | NO | YES | NO | YES | NO | YES | NR | YES | NO | FAIR |
| Bhalla et al. (2010) | YES | NO | YES | NO | YES | YES | YES | NR | YES | YES | FAIR |
| Borghi et al. (2016) | YES | YES | YES | YES | YES | YES | YES | NR | YES | NO | GOOD |
| Castro et al. (2016) | YES | YES | YES | NO | YES | YES | YES | NR | YES | YES | GOOD |
| Chawda et al. (2010) | YES | YES | YES | NO | YES | YES | YES | NR | YES | YES | GOOD |
| Colombo et al. (2016a) | YES | YES | YES | NO | YES | YES | YES | NR | YES | NO | FAIR |
| Colombo et al. (2016b) | YES | YES | YES | NO | YES | YES | YES | NR | YES | NO | FAIR |
| de Farias; Bezerra (2003) | YES | YES | YES | NO | YES | NO | YES | NR | YES | YES | FAIR |
| Davidopoulou et al. (2012) | YES | YES | YES | NO | YES | YES | YES | NR | YES | YES | GOOD |
| Doifode; Damle (2011) | YES | NO | YES | NO | YES | YES | YES | NR | YES | NO | FAIR |
| Doods et al. (1997) | YES | YES | YES | NO | YES | YES | YES | NR | YES | YES | GOOD |
| Felizardo et al. (2010) | YES | YES | YES | NO | YES | YES | YES | NR | YES | YES | GOOD |
| Frasseto et al. (2012) | YES | YES | YES | NO | YES | YES | YES | NR | YES | NO | FAIR |
| Hedenbjörk-Lager et al. (2015) | YES | YES | NO | NO | YES | NO | YES | NR | YES | NO | FAIR |
| Hedge et al. (2009) | NO | NO | YES | NO | YES | YES | YES | NR | YES | NO | FAIR |
| Hedge et al.(2013a) | YES | NO | YES | NO | YES | YES | YES | NR | YES | NO | FAIR |
| Hegde et al.(2013b) | YES | NO | YES | NO | YES | YES | YES | NR | YES | NO | FAIR |
| Hegde et al. (2013c) | YES | NO | YES | NO | YES | YES | YES | NR | YES | YES | FAIR |
| Hegde et al. (2014) | YES | NO | YES | NO | YES | YES | YES | NR | YES | YES | FAIR |
| Jurczak et al. (2015) | YES | NO | YES | NO | YES | YES | YES | NR | YES | YES | FAIR |
| Jurczak et al. (2017) | YES | YES | YES | NO | YES | YES | YES | NR | YES | YES | GOOD |
| Karthika et al. (2021) | YES | YES | YES | YES | YES | NO | YES | NR | YES | NO | FAIR |
| Koopaie et al. (2021) | YES | YES | YES | NO | YES | YES | YES | NR | YES | NO | FAIR |
| Lamberts et al. (1984) | YES | YES | NR | NO | YES | NO | YES | NR | YES | NO | FAIR |
| Letieri et al. ( 2019) | YES | NO | YES | NO | YES | YES | YES | NR | YES | YES | FAIR |
| Lertsirivorakul et al. (2015) | YES | YES | YES | NO | YES | YES | YES | NR | YES | NO | FAIR |
| Luthfi et al. (2019) | YES | YES | YES | NO | YES | YES | YES | NR | YES | NO | FAIR |
| Mahjoub et al.(2014) | YES | YES | YES | NO | YES | YES | YES | NR | YES | YES | GOOD |
| Mandel et al. (1965) | NO | YES | NO | NO | YES | NO | YES | NR | YES | NO | POOR |
| Mandel et al. (1983) | YES | YES | YES | NO | YES | NO | YES | NR | YES | NO | FAIR |
| Mojarad et al. (2013) | YES | YES | YES | YES | YES | NO | YES | NR | YES | YES | GOOD |
| Moncada et al.(2015) | YES | YES | YES | YES | YES | YES | YES | NR | YES | NO | GOOD |
| Moslemi et al (2015) | YES | YES | YES | YES | YES | YES | YES | NR | YES | NO | GOOD |
| Muchndi et al. (2015) | YES | NO | YES | NO | YES | YES | YES | NR | YES | NO | FAIR |
| Murugeshappa et al.( 2018) | YES | YES | YES | NO | YES | YES | YES | NR | YES | YES | GOOD |
| Nascimento et al. (2009) | YES | YES | YES | NO | YES | NO | YES | NR | YES | YES | FAIR |
| Nascimento et al. (2013) | YES | YES | YES | NO | YES | YES | YES | NR | YES | YES | GOOD |
| Naspitz et al. (1999) | YES | NO | NO | NO | YES | NO | YES | NR | YES | NO | POOR |
| Nireeksha et al. (2017) | YES | NO | YES | NO | YES | NO | YES | NR | YES | YES | FAIR |
| Öztürk et al.(2008) | YES | YES | YES | YES | YES | YES | YES | NR | YES | YES | FAIR |
| Pandey et al. (2015) | YES | YES | YES | NO | YES | YES | YES | NR | YES | YES | GOOD |
| Pandey et al. (2018) | YES | YES | NO | NO | YES | YES | YES | NR | YES | YES | FAIR |
| Patel; Pujara (2015) | YES | YES | YES | NO | YES | YES | YES | NR | YES | NO | FAIR |
| Phattarataratip et al. (2011) | YES | YES | NO | NO | YES | NO | YES | YES | YES | NO | FAIR |
| Picco et al.(2017) | YES | NO | YES | YES | YES | YES | YES | NR | YES | NO | FAIR |
| Picco et al. (2019) | YES | NO | YES | YES | YES | YES | YES | NR | YES | NO | FAIR |
| Prabhakar; Shubha; Mahantesh (2008) | YES | YES | YES | NO | YES | NO | YES | NR | YES | YES | FAIR |
| Preethi et el.(2010) | YES | NO | YES | NO | YES | YES | YES | NR | YES | NO | FAIR |
| Priya et al. (2013) | YES | NO | YES | NO | YES | YES | YES | NR | YES | YES | FAIR |
| Pyati et al.(2018) | YES | YES | YES | YES | YES | YES | YES | NR | YES | YES | GOOD |
| Rahmani et al. (2016) | YES | YES | YES | NO | YES | YES | YES | NR | YES | YES | GOOD |
| Ranadheer et al. (2011) | YES | NO | YES | NO | YES | YES | YES | NR | YES | NO | FAIR |
| Razi et al. (2020) | YES | YES | YES | NO | YES | YES | YES | NR | YES | YES | GOOD |
| Reyes et al. (2014) | YES | YES | YES | YES | YES | NO | YES | NR | YES | YES | GOOD |
| Roa et al. (2008) | YES | YES | YES | NO | YES | YES | YES | NR | YES | NO | FAIR |
| Salman et al. (2021) | YES | YES | YES | NO | YES | NO | YES | NR | YES | NO | FAIR |
| Shaki et al. (2020) | YES | NO | YES | NO | YES | YES | YES | NR | YES | YES | FAIR |
| Shifa et al. (2008) | YES | NO | YES | NO | YES | NO | YES | YES | YES | YES | FAIR |
| Shu et al. (2007) | YES | NO | YES | NO | YES | NO | YES | NR | YES | YES | FAIR |
| Silva et al. (2016) | YES | YES | YES | NO | YES | YES | YES | NR | YES | YES | GOOD |
| Singh et al.(2015) | YES | YES | NO | NO | YES | YES | YES | NR | YES | NO | FAIR |
| Sousa et al. (2020) | YES | YES | YES | YES | YES | YES | YES | NR | YES | YES | GOOD |
| Stuchell & Mandel (1978) | YES | NO | NO | NO | YES | NO | YES | NR | YES | NO | POOR |
| Stuchell; Mandel (1983) | YES | NO | YES | NO | YES | NO | YES | NR | YES | NO | FAIR |
| Szabó, (1974) | NO | YES | NO | NO | YES | NO | YES | NR | YES | NO | POOR |
| Szkaradkiewcz-Karpinska et al. (2017) | YES | YES | YES | NO | YES | YES | YES | NR | YES | NO | FAIR |
| Szkaradkiewicz-Karpinska et al. (2019) | YES | YES | YES | NO | YES | YES | YES | NR | YES | YES | GOOD |
| Tao et al. (2005) | YES | YES | NO | NO | YES | NO | YES | NR | YES | NO | FAIR |
| Thaweboon et al. (2008) | YES | YES | YES | NO | YES | NO | YES | NR | YES | YES | FAIR |
| Toomarian et al. (2011) | YES | YES | YES | NO | YES | YES | YES | NR | YES | NO | FAIR |
| Tulunoglu et al. (2006) | YES | YES | YES | NO | YES | YES | YES | NR | YES | NO | GOOD |
| VanWuyckhuysel et al. (1995) | YES | YES | NO | NO | YES | YES | YES | NR | YES | YES | FAIR |
| Vieira et al. (2020) | YES | YES | NR | YES | YES | NR | YES | NR | YES | NO | FAIR |
| Yang et al. (2015a) | YES | YES | YES | NO | YES | YES | YES | NR | YES | YES | GOOD |
| Yang et al. (2015b) | YES | YES | NO | NO | YES | YES | YES | NR | YES | NO | FAIR |
| Zengo et al.(1971) | YES | NO | NO | NO | YES | NO | YES | NR | YES | NO | POOR |

Q1. Was the research question or objective in this paper clearly stated?

Q2. Was the study population clearly specified and defined?

Q3. Were inclusion and exclusion criteria for being in the study pre-specified and applied uniformly to all participants?

Q4. Was a sample size justification, power description, or variance and effect estimates provided?

Q5. For exposures that can vary in amount or level, did the study examine different levels of the exposure as related to the outcome?

Q6. Were the outcome measures (dependent variables) clearly defined, valid, reliable, and implemented consistently across all study participants?

Q7. Were the outcome measures (dependent variables) clearly defined, valid, reliable, and implemented consistently across all study participants?

Q8. Were the outcome assessors blinded to the exposure status of participants?

Q9. Were key potential confounding variables measured and adjusted statistically for their impact on the relationship between exposure(s) and outcome(s)?

Q10 - Clear criteria for sample collection (Biofim/saliva)?

Green, Yellow and Red colors mean that the overall quality of the study was good, fair or poor, respectively.
