# Supplementary material for: Comparison of oral cavity protein abundance among caries-free and caries-affected individuals—a systematic review and meta-analysis
Source: Front Oral Health. 2023 Sep 15;4:1265817. doi: 10.3389/froh.2023.1265817 (PMC10540632; doi:10.3389/froh.2023.1265817)
Supplement: Supplementary file 14 [file Datasheet1.pdf]

Comparison of oral cavity protein abundance among caries-free and caries-affected individuals – a systematic review and meta-analysis

Eliane Garcia da Silveira[1,2], Laura Schaurich Prato[3], Sarah Freygang Mendes Pilati[4], Rodrigo Alex Arthur[5*].

[1]Department of Preventive and Community Dentistry, Federal University of Rio Grande do Sul, Porto Alegre/RS; Brazil

[2]University of Vale do Itajai, Itajaí/SC; Brazil

[3]Department of Preventive and Community Dentistry, Federal University of Rio Grande do Sul, Porto Alegre/RS; Brazil

[4]University of Vale do Itajai, Itajaí/SC; Brazil

[5]Department of Preventive and Community Dentistry, Federal University of Rio Grande do Sul, Porto Alegre/RS; Brazil

*Correspondence:

Rodrigo Alex Arthur

Preventive and Community Dentistry

Federal University of Rio Grande do Sul (UFRGS)

R. Ramiro Barcelos, 2492

Porto Alegre, Rio Grande do Sul, CEP 90035-003, Brasil

Tel: +55 51 3308 5193

E-mail: rodrigoarthur.ufrgs@gmail.com

Supplementary Figures

Study or Subgroup	Dental Caries Mean	Dental Caries SD	Dental Caries Total	Caries-Free Mean	Caries-Free SD	Caries-Free Total	Weight	Std. mean difference IV, Random, 95% CI
Hedge et al. 2013	1273.64	423	60	1357.11	422.88	20	33.8%	-0.20 [-0.70 , 0.31]
Bagherian et al. 2018	9.78	3.26	45	8.49	2.75	45	36.3%	0.42 [0.01 , 0.84]
Razi et al. 2020	0.87	0.14	20	1.04	0.31	20	29.9%	-0.69 [-1.33 , -0.05]
**Total (95% CI)**			**125**			**85**	**100.0%**	**-0.12 [-0.75 , 0.51]**

Heterogeneity: Tau² = 0.24; Chi² = 9.02, df = 2 (P = 0.01); I² = 78%
Test for overall effect: Z = 0.37 (P = 0.71)
Test for subgroup differences: Not applicable

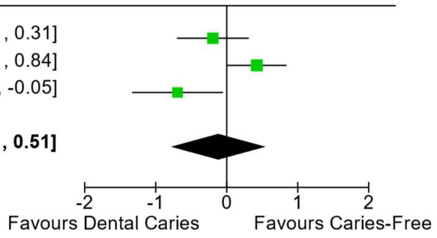

Figure S1. Standardized mean difference (SMD) and confidence intervals for IgG concentration (mg/dL) in the oral cavity of caries-free and caries-affected individuals. Positive SMD values mean lower IgG concentration in caries-free individuals (favours caries-free). Negative SMD values mean lower IgG concentration in caries-affected individuals (favours dental caries)

Study or Subgroup	Dental Caries Mean	Dental Caries SD	Dental Caries Total	Caries-free Mean	Caries-free SD	Caries-free Total	Weight	Std. mean difference IV, Random, 95% CI
Prabhakar et al. 2008	69600	17084.09	40	85000	23100	20	22.9%	-0.79 [-1.35 , -0.23]
de Farias and Bezerra 2013	122.8	101.5	20	71.9	48.6	20	20.9%	0.63 [-0.01 , 1.26]
Mojarad et al. 2013	0.2827	0.2173	84	0.4208	0.2725	84	29.3%	-0.56 [-0.87 , -0.25]
Ahmad et al. 2021	68420	26280	50	83530	27610	50	27.0%	-0.56 [-0.96 , -0.16]
**Total (95% CI)**			**194**			**174**	**100.0%**	**-0.36 [-0.84 , 0.11]**

Heterogeneity: Tau² = 0.18; Chi² = 13.16, df = 3 (P = 0.004); I² = 77%
Test for overall effect: Z = 1.50 (P = 0.13)
Test for subgroup differences: Not applicable

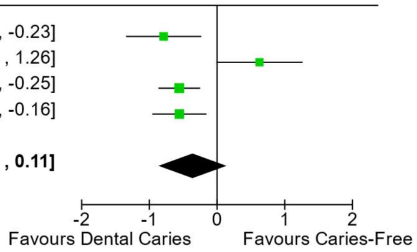

Figure S2. Standardized mean difference (SMD) and confidence intervals for salivary alpha-amylase levels (U/L) in the oral cavity of caries-free and caries-affected individuals. Positive SMD values mean lower alpha-amylase activity in caries-free individuals (favours caries-free). Negative SMD values mean lower alpha-amylase activity in caries-affected individuals (favours dental caries)

	Dental Caries			Caries-Free				Std. mean difference
Study or Subgroup	Mean	SD	Total	Mean	SD	Total	Weight	IV, Random, 95% CI
Phattarataratip et al. 2011	15.92	7.5	30	15.7	12.86	30	40.6%	0.02 [-0.49 , 0.53]
Al-Ali et al. 2021	1.83	1.861	44	2.179	1.883	44	59.4%	-0.18 [-0.60 , 0.23]
**Total (95% CI)**			**74**			**74**	**100.0%**	**-0.10 [-0.42 , 0.22]**

Heterogeneity: $Tau^2 = 0.00$; $Chi^2 = 0.38$, df = 1 (P = 0.54); $I^2 = 0\%$
Test for overall effect: Z = 0.62 (P = 0.54)
Test for subgroup differences: Not applicable

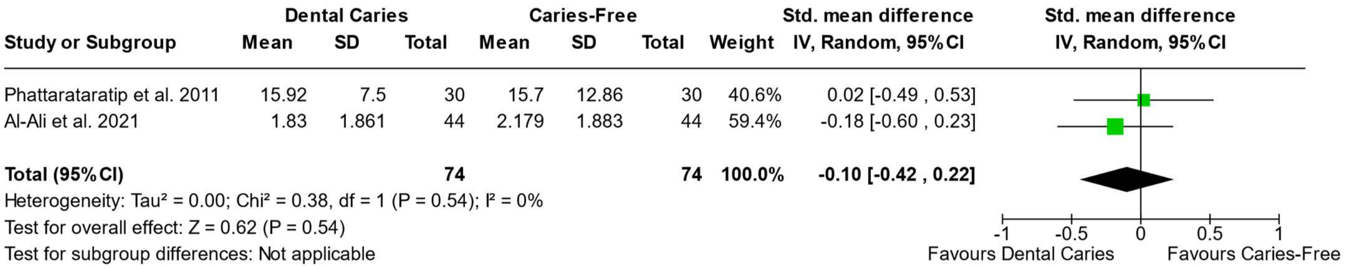

Figure S3. Standardized mean difference (SMD) and confidence intervals for LL-37 concentration (ng/mL) in the oral cavity of caries-free and caries-affected individuals. Positive SMD values mean lower LL-37 concentration in caries-free individuals (favours caries-free). Negative SMD values mean lower LL-37 concentration in caries-affected individuals (favours dental caries)

	Dental Caries			Caries-Free				Std. mean difference
Study or Subgroup	Mean	SD	Total	Mean	SD	Total	Weight	IV, Random, 95% CI
Phattarataratip et al. 2011 (hBD-2)	0.0021	0.0019	30	0.0023	0.0024	30	24.8%	-0.09 [-0.60 , 0.42]
Phattarataratip et al. 2011 (hBD-3)	0.00086	0.0009	30	0.00085	0.00083	30	24.8%	0.01 [-0.49 , 0.52]
Jurczak et al. 2015 (hBD-2)	0.0022	0.00005	41	0.0021	0.00007	41	24.9%	1.63 [1.13 , 2.13]
Al-Ali et al. 2021 (hBD)	176.49	175.93	44	275.35	256.56	44	25.4%	-0.45 [-0.87 , -0.02]
**Total (95% CI)**			**145**			**145**	**100.0%**	**0.27 [-0.63 , 1.17]**

Heterogeneity: $Tau^2 = 0.78$; $Chi^2 = 41.76$, df = 3 (P < 0.00001); $I^2 = 93\%$
Test for overall effect: Z = 0.59 (P = 0.55)
Test for subgroup differences: Not applicable

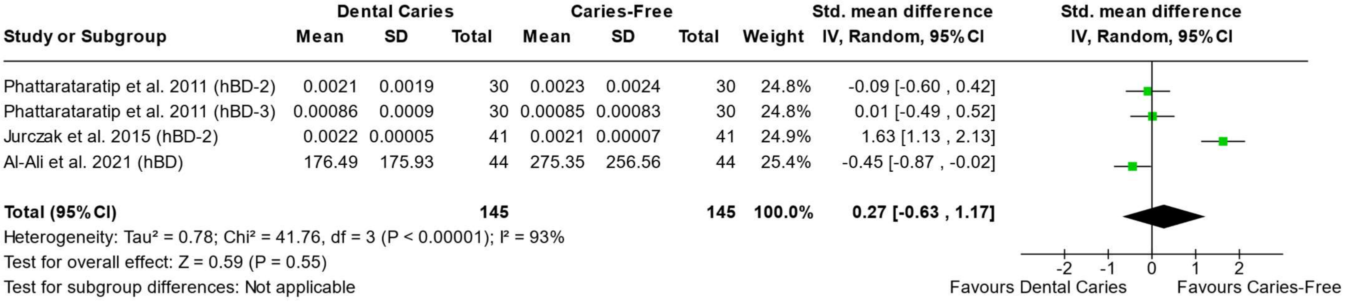

Figure S4. Standardized mean difference (SMD) and confidence intervals for hBD concentration (ug/mL) in the oral cavity of caries-free and caries-affected individuals. Positive SMD values mean lower hBD concentration in caries-free individuals (favours caries-free). Negative SMD values mean lower hBD concentration in caries-affected individuals (favours dental caries)

Study or Subgroup	Dental Caries Mean	Dental Caries SD	Dental Caries Total	Caries-Free Mean	Caries-Free SD	Caries-Free Total	Weight	Std. mean difference IV, Random, 95% CI
Phattarataratip et al. 2011 (hNP 1-3)	2.047	1.254	30	1.779	1.055	30	34.8%	0.23 [-0.28 , 0.74]
Toomarian et al. 2011 (hNP 1-3)	0.086	0.083	60	0.084	0.017	27	38.2%	0.03 [-0.43 , 0.48]
Luthfi et al. 2019 (hNP 1-3)	0.000172	0.000041	20	0.00014	0.000031	20	27.1%	0.86 [0.21 , 1.51]
**Total (95% CI)**			**110**			**77**	**100.0%**	**0.32 [-0.13 , 0.77]**

Heterogeneity: Tau² = 0.08; Chi² = 4.30, df = 2 (P = 0.12); I² = 53%
Test for overall effect: Z = 1.41 (P = 0.16)
Test for subgroup differences: Not applicable

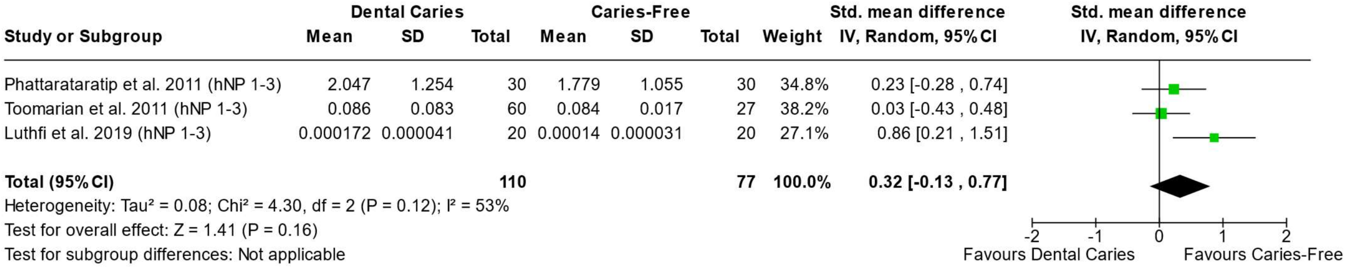

Figure S5. Standardized mean difference (SMD) and confidence intervals for hNP concentration (ug/mL) in the oral cavity of caries-free and caries-affected individuals. Positive SMD values mean lower hNP concentration in caries-free individuals (favours dental caries).
